# mTORC1 is a mechanosensor that regulates surfactant function and lung compliance during ventilator-induced lung injury

**DOI:** 10.1172/jci.insight.137708

**Published:** 2021-07-22

**Authors:** Hyunwook Lee, Qinqin Fei, Adam Streicher, Wenjuan Zhang, Colleen Isabelle, Pragi Patel, Hilaire C. Lam, Antonio Arciniegas-Rubio, Miguel Pinilla-Vera, Diana P. Amador-Munoz, Diana Barragan-Bradford, Angelica Higuera-Moreno, Rachel K. Putman, Lynette M. Sholl, Elizabeth P. Henske, Christopher M. Bobba, Natalia Higuita-Castro, Emily M. Shalosky, R. Duncan Hite, John W. Christman, Samir N. Ghadiali, Rebecca M. Baron, Joshua A. Englert

**Affiliations:** 1 Division of Pulmonary, Critical Care, and Sleep Medicine, Department of Internal Medicine, and; 2 The Dorothy M. Davis Heart and Lung Research Institute, The Ohio State Wexner Medical Center, Columbus, Ohio, USA.; 3 Division of Pulmonary and Critical Care Medicine, Department of Medicine, and; 4 Department of Pathology, Brigham and Women’s Hospital, Harvard Medical School, Boston, Massachusetts, USA.; 5 Department of Biomedical Engineering, The Ohio State University, Columbus, Ohio, USA.; 6 Division of Pulmonary, Critical Care, and Sleep Medicine, The University of Cincinnati College of Medicine, Cincinnati, Ohio, USA.

**Keywords:** Pulmonology, Pulmonary surfactants, Signal transduction

## Abstract

The acute respiratory distress syndrome (ARDS) is a highly lethal condition that impairs lung function and causes respiratory failure. Mechanical ventilation (MV) maintains gas exchange in patients with ARDS but exposes lung cells to physical forces that exacerbate injury. Our data demonstrate that mTOR complex 1 (mTORC1) is a mechanosensor in lung epithelial cells and that activation of this pathway during MV impairs lung function. We found that mTORC1 is activated in lung epithelial cells following volutrauma and atelectrauma in mice and humanized in vitro models of the lung microenvironment. mTORC1 is also activated in lung tissue of mechanically ventilated patients with ARDS. Deletion of *Tsc2*, a negative regulator of mTORC1, in epithelial cells impairs lung compliance during MV. Conversely, treatment with rapamycin at the time MV is initiated improves lung compliance without altering lung inflammation or barrier permeability. mTORC1 inhibition mitigates physiologic lung injury by preventing surfactant dysfunction during MV. Our data demonstrate that, in contrast to canonical mTORC1 activation under favorable growth conditions, activation of mTORC1 during MV exacerbates lung injury and inhibition of this pathway may be a novel therapeutic target to mitigate ventilator-induced lung injury during ARDS.

## Graphical abstract

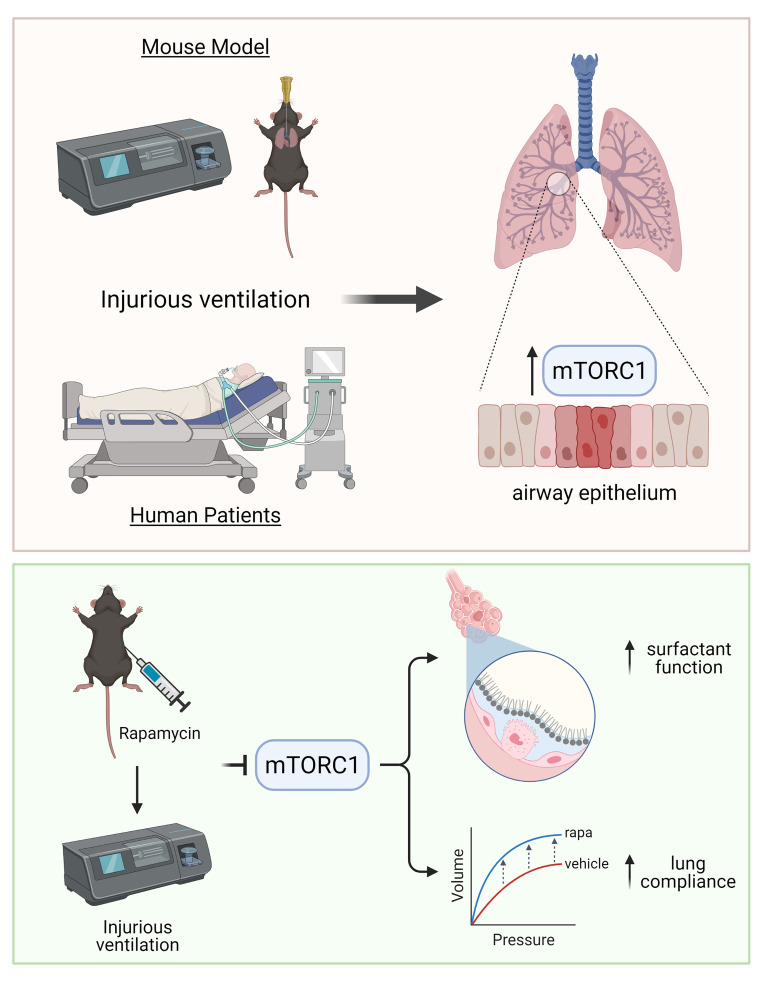

## Introduction

The acute respiratory distress syndrome (ARDS) is a devastating condition that affected over 200,000 patients annually in the US prior to COVID-19 with millions of cases worldwide during the pandemic (1–3). ARDS is associated with mortality rates of up to 45% in its most severe form (4). The only treatment for patients with ARDS is supportive care with mechanical ventilation (MV), and although life-saving, MV can exacerbate existing lung injury and even cause de novo injury, known as ventilator-induced lung injury (VILI) (5). Limiting lung distention by using low tidal volume (TV) ventilation decreases mortality in patients with ARDS (6), but factors such as regional heterogeneity lead to persistent injury even with low TV ventilation (7). VILI arises from three injurious forces, including excessive stretch (volutrauma), increased transmural pressure (barotrauma), and mechanical stress from repetitive collapse and reopening of lung units (atelectrauma) (8). The molecular mechanisms by which these injurious forces cause lung injury remain poorly understood, which has limited the development of targeted therapies to prevent lung injury in patients requiring MV, including patients with ARDS.

Mechanotransduction is the process by which physical forces are transduced into biologic responses. The lungs stretch cyclically during spontaneous breathing and with positive pressure during MV. The precise mechanisms by which the lungs sense and respond to injurious forces during MV are not well understood. Several cell types have been implicated in the transduction of mechanical signals following injurious ventilation, including epithelial (9, 10) and endothelial cells (11). One approach to elucidate the molecular mechanisms of lung injury during MV is to identify mechanosensitive signaling pathways that are differentially activated by the various types of VILI.

mTOR complex 1 (mTORC1) is a ubiquitously expressed multi-protein complex with serine/threonine kinase activity that is involved in cellular metabolism and response to stress. mTORC1 activation has been also been implicated in mechanotransduction in muscles (12–14). In the lungs, mTORC1 contributes to the pathogenesis of lung injury in response to cigarette smoke (15) and endotoxins (16). However the role of mTORC1 activation in response to mechanical forces in the lung and its role in the pathogenesis of VILI has not been examined. In contrast to canonical activation of mTORC1 under favorable growth conditions, we discovered that mTORC1 is pathologically activated by injurious forces during MV. Based on this observation, we investigated whether mTORC1 activation mediates the development of lung injury following injurious MV and whether inhibition of this pathway might represent a novel therapeutic target for patients with ARDS.

## Results

### Volutrauma and atelectrauma activate mTORC1 in epithelial cells in murine models of injurious MV.

**Figure 1:**
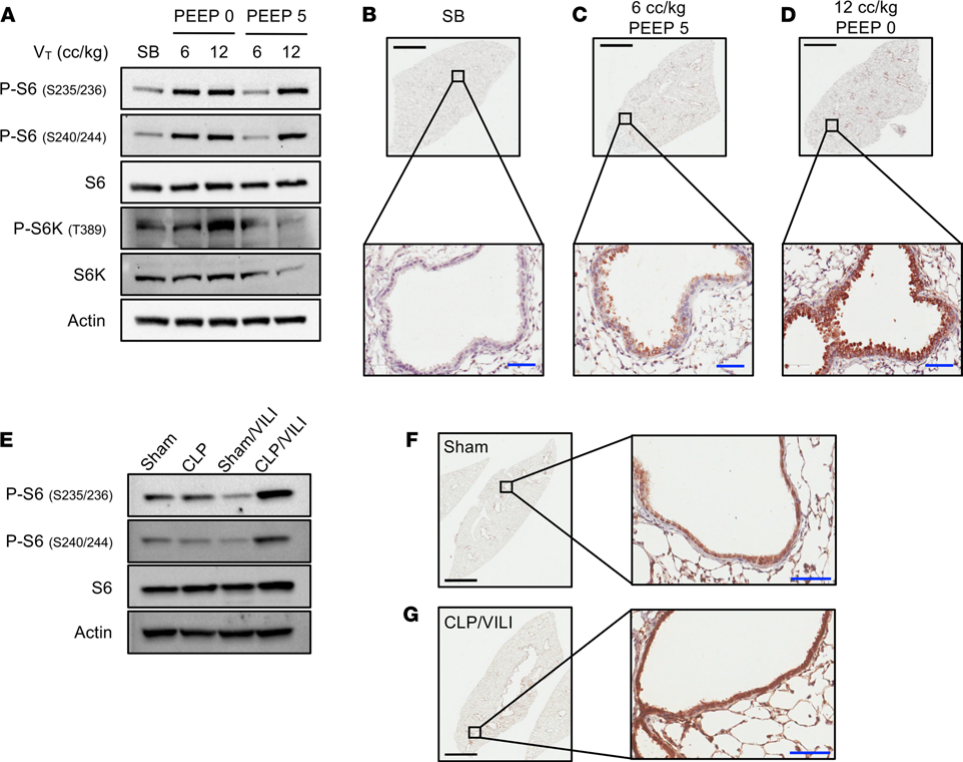
Injurious MV activates mTORC1 in lung epithelial cells. (**A**) Immunoblots of phosphorylated and total ribosomal S6, S6 kinase (S6K), and beta-actin using pooled protein lysate from whole lung tissue of SB control mice (*n* = 3/lane) or mice subjected to MV (*n* = 4/lane) with high TV (V_T_, 12 cc/kg), low TV (V_T_ 6 cc/kg), with or without the use of PEEP (0 or 5 cm H_2_O). Low power (4×) and high power (400×, inset) images from lung tissue that was immunostained for phosphorylated S6 (P-S6, Ser235/236) from SB control mice (**B**), mice ventilated with noninjurious settings (**C**) (V_T_ 6 cc/kg, PEEP 5 cm H_2_O), and mice ventilated with injurious settings (**D**) (V_T_ 12 cc/kg, PEEP 0 cm H_2_O). (**E**) Immunoblots from whole lung tissue of mice subjected to sham laparotomy (*n* = 3/lane), CLP,(*n* = 4/lane), and VILI (V_T_ 12 cc/kg, PEEP 2.5 cm H_2_O) 24 hours after sham laparotomy (*n* = 3/lane) and VILI 24 hours after CLP (CLP/VILI, *n* = 4/lane). Representative images from lung tissue that was immunostained for P-S6 (Ser235/236) from sham (**F**) and CLP/VILI (**G**) mice. Scale bars: black bars = 2 mm, blue bars = 50 μm.

We used a model of simultaneous volutrauma (TV [V_T_, 12 cc/kg]) and atelectrauma positive end expiratory pressure (PEEP; 0 cm H_2_O) that impaired lung function (i.e., decreased compliance), induced inflammation (i.e., bronchoalveolar lavage [BAL] neutrophils and IL-6 levels), and increased barrier permeability (i.e., BAL protein levels) (Supplemental Figure 1; supplemental material available online with this article; https://doi.org/10.1172/jci.insight.137708DS1). mTORC1 activation was assessed in lung tissue by immunoblotting for phosphorylated isoforms of the ribosomal S6 protein (17, 18) and S6 kinase (19). Spontaneously breathing (SB) control mice and mice ventilated with noninjurious settings (V_T_ 6 cc/kg, PEEP 5 cm H_2_O) had low levels of mTORC1 activation.. In contrast, mice ventilated with injurious high TVs (12 cc/kg) or without PEEP had increased levels of phosphorylated S6 (Figure 1A). Immunostaining revealed that mTORC1 was activated in multiple cell types, including alveolar epithelial cells with a type II morphology and alveolar macrophages, but the most intense site of activation was the airway epithelium (Figure 1, B–D, and Supplemental Figure 2). Specifically, there were high levels of P-S6 in CC10 expressing cells following VILI (Supplemental Figure 3). Given that critically ill patients frequently develop ARDS and require MV in the setting of the existing lung injury, we also subjected mice to a 2-hit model of ARDS in which MV was initiated in the setting of polymicrobial sepsis induced by cecal ligation and puncture (CLP). Twenty-four hours after sublethal CLP or sham operation, mice were subjected to injurious MV (CLP/VILI-12 cc/kg, PEEP 2.5 cm H_2_O) for 4 hours. Mice subjected to CLP/VILI had decreased lung compliance and increased markers of lung injury compared with uninjured control mice or mice that had either injury alone (Supplemental Figure 4), which correlated with increased mTORC1 activation in whole lung tissue (Figure 1E). As was the case with VILI alone, the most prominent site of mTORC1 activation following CLP/VILI was the epithelium (Figure 1, F and G). These data demonstrate that mTORC1 is activated in lung epithelial cells following injurious MV.

### Tsc2 deletion in airway epithelial cells impairs lung function during MV.

**Figure 2:**
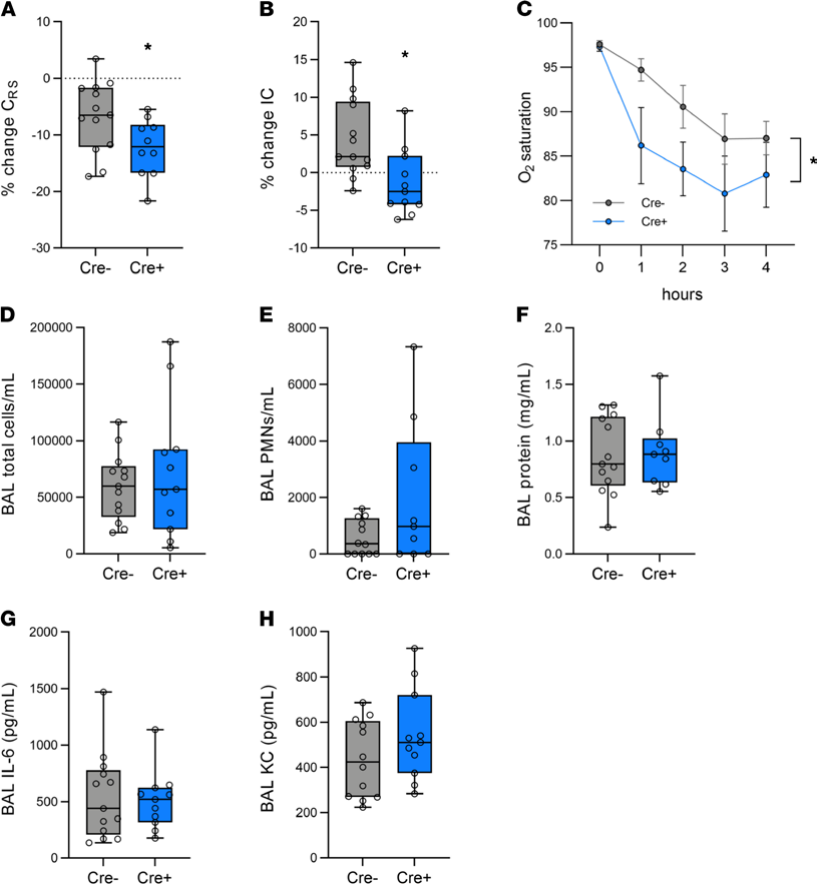
Airway epithelial *Tsc2* deletion impairs lung function in a murine model of combined volutrauma and atelectrauma. Mice with airway epithelial *Tsc2* deletion (Cre+) and Cre– control mice were subjected to MV with high TV (12 cc/kg) without PEEP (0 cm H_2_O) for 4 hours. (**A**) The change in respiratory system compliance (C_RS_) and (**B**) inspiratory capacity (IC) were measured during MV. All data were normally distributed, analyzed by Student’s *t* test, *P < 0.05. (**C**) Oxygen saturations measured via pulse oximetry during the 4-hour period of ventilation presented as mean ± SEM. *P < 0.05, by 2-way ANOVA with repeated measures with Holms-Sidak post hoc test. Following MV, a bronchoalveolar lavage (BAL) was performed and total inflammatory cells (**D**), neutrophils (PMNs) (**E**), and protein levels (**F**) were measured. For PMN counts, *n* = 12 for Cre- and *n* = 9 for Cre+. IL-6 (**G**) and KC (**H**) levels were measured in BAL fluid by ELISA. *n* = 13 for Cre- and *n* = 11 for Cre+ unless otherwise noted. Box blots show median ± interquartile range and whiskers define min and max values.

To determine whether mTORC1 activation in lung epithelial cells mediates the development of lung injury or represents a compensatory response that promotes recovery, we used a genetic approach to generate mice with increased mTORC1 activation in CC10 expressing airway epithelial cells (Supplemental Figure 3). We bred mice with homozygously floxed alleles of *Tsc2* (a negative regulator of mTORC1) (20) to mice expressing Cre recombinase under the control of the airway epithelial specific CC10 promoter (21). CC10-Cre mice were bred to mT/mG reporter mice (22) to confirm airway epithelial specific expression of the Cre recombinase prior to breeding with *Tsc2* flox mice (Supplemental Figure 5). *CC10*^Cre^/*Tsc2*^flox/flox^ progeny were backcrossed to a C57BL/6 background and an examination of lung morphology revealed no abnormalities at baseline (Supplemental Figure 5). Lung compliance was not affected by *Tsc2* deletion or the expression of Cre recombinase (Supplemental Figure 5). Airway resistance was also normal in mice with airway epithelial *Tsc2* deletion (Supplemental Figure 5). There was an increase in the total number of BAL cells (Supplemental Figure 5) but more than 95% of cells were alveolar macrophages and there was no increase in BAL neutrophils (data not shown). Low levels of mTORC1 activation (i.e., P-S6) were observed in (SB) Cre– control mice. Mice with airway epithelial *Tsc2* deletion (Cre+) mice had increased mTORC1 activation in airway epithelial cells by immunohistochemical staining (Supplemental Figure 5). Primary tracheobronchial epithelial cells (23) isolated from Cre+ mice had decreased tuberin (encoded by *Tsc2*) expression and increased S6 phosphorylation (Supplemental Figure 5). Cre+ mice were subjected to simultaneous volutrauma and atelectrauma (12 cc/kg, PEEP 0 cm H_2_O) for 4 hours and had decreased respiratory system compliance and decreased inspiratory capacity compared with Cre– controls (Figure 2, A and B). Cre+ mice were also more hypoxemic during injurious ventilation (Figure 2C). Notably, recruitment of inflammatory cells, barrier permeability (BAL protein), and proinflammatory BAL cytokine and chemokine levels were not different following VILI or in SB control mice (Figure 2, D–H, and Supplemental Figure 6). These data demonstrate that airway epithelial *Tsc2* deletion increases mTORC1 activation and exacerbates physiologic lung injury (i.e., decreases lung compliance, decreases inspiratory capacity, and impairs oxygenation) following injurious ventilation without altering lung inflammation or barrier dysfunction.

### Mechanically ventilated patients with diffuse alveolar damage have increased mTORC1 activation in lung tissue.

**Figure 3:**
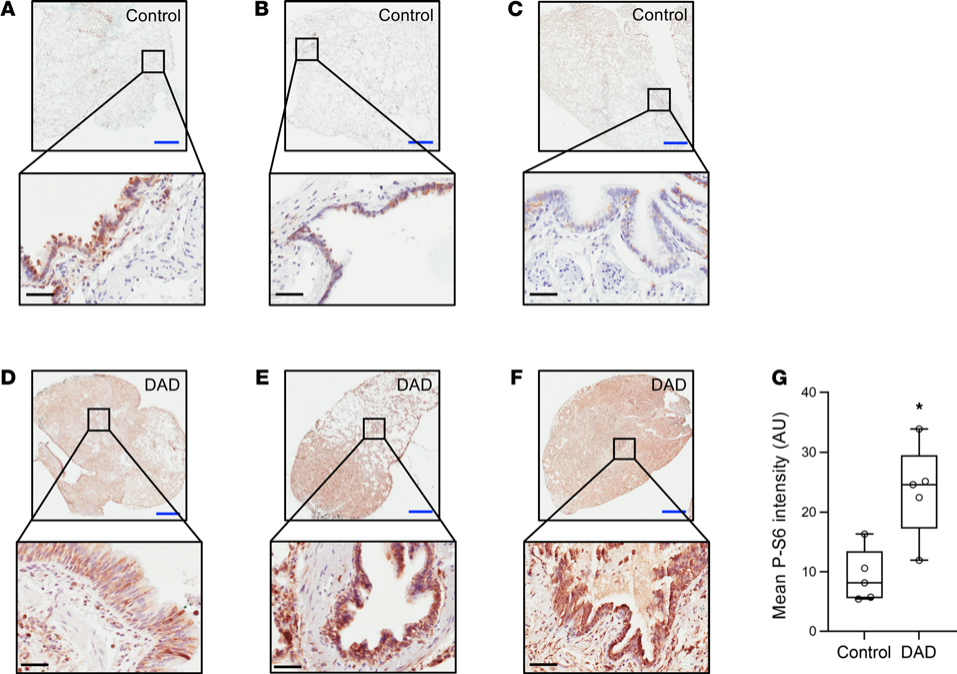
mTORC1 is activated in lung tissue from mechanically ventilated patients with DAD. Photomicrographs of lung sections stained for P-S6 (Ser235/236) from normal lung tissue from control patients (**A**–**C**) and from patients with DAD (**D**–**F**). Mean intensity of staining was quantitated using low power images of the entire slide (**G**) (*n* = 5/group). Scale bars: blue bar = 2 mm, black bar = 50 μm. *P < 0.05 versus control by Student’s *t* test. Box blots show median ± interquartile range and whiskers define min and max values.

To assess whether mTORC1 is activated in patients with ARDS undergoing MV, we obtained formalin-fixed lung tissue from a pathology tissue bank. Specimens were analyzed from 5 patients with diffuse alveolar damage (DAD), the most common lung pathology in patients with ARDS (4, 24). Normal lung tissue adjacent to resected lung tumors from 5 subjects was used as a control. Clinical characteristics of the subjects are shown in Supplemental Table 1, and there were no significant differences between groups. Notably, all patients were receiving MV at the time of biopsy. Immunostaining for phosphorylated ribosomal S6 revealed increased mTORC1 activation in lung tissue from patients with DAD compared with normal lung tissue (Figure 3, A–F, and Supplemental Figure 7). Although diffuse staining was present throughout the lung, it appeared that the most prominent site of staining was the epithelium, including airway epithelial cells (Figure 3, D–F). The intensity of P-S6 staining was quantitated by image analysis and was significantly higher in patients with DAD compared with controls (Figure 3G). To determine whether mTORC1 activation was due to the underlying lung injury or the use of MV, we obtained additional biopsies from SB patients undergoing transbronchial biopsies during bronchoscopy or from surgical wedge resections from mechanically ventilated patients. Biopsies had a variety of pathologic findings including several with DAD, several types of interstitial lung disease, airway diseases, and no specific pathology. In general, SB patients had less mTORC1 activation compared with mechanically ventilated patients regardless of the underlying pathology (Supplemental Figure 8). Patients without lung injury had low levels of mTORC1 activation (Supplemental Figure 8). These data suggest that injurious MV in the context of underlying lung injury activates mTORC1 in the lung epithelium.

### In vitro models of volutrauma and atelectrauma activate mTORC1 in primary human airway epithelial cells.

**Figure 4:**
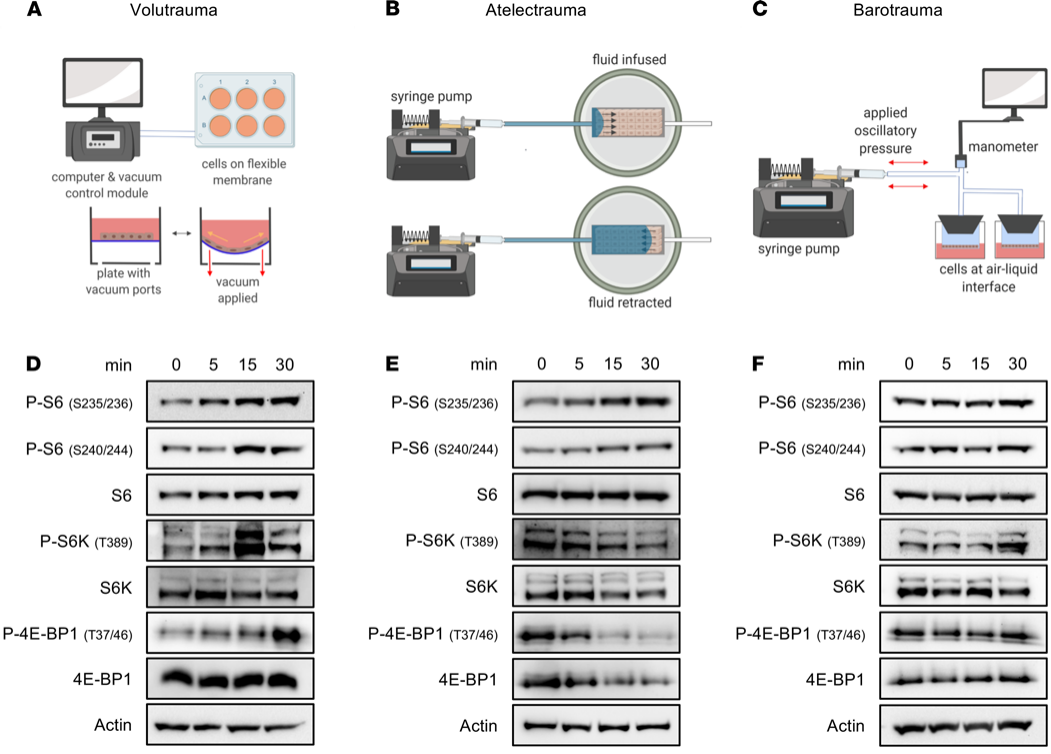
mTORC1 is rapidly activated by volutrauma and atelectrauma in vitro. Schematics of in vitro volutrauma, atelectrauma, and barotrauma models used (**A**–**D**). SAECs were subjected to equibiaxial stretch (20%, 0.2 Hz) for varying amounts of time prior to immunoblotting for markers of mTORC1 activation (protein pooled from *n* = 3 wells/lane). (**E**) SAECs were grown to confluent monolayers on collagen-coated glass slides in a microfluidic chamber and subjected to bubble flow (velocity 30 mm/sec) to model atelectrauma for varying amounts of time prior to immunoblotting for markers of mTORC1 activation (protein pooled from *n* = 2 gels/time point). (**F**) SAECs were grown at air-liquid interface on Transwells and subjected to oscillatory pressure (30 cm H_2_O, 0.2 Hz) for varying amounts of time prior to immunoblotting for markers of mTORC1 activation (protein pooled from *n* = 3 wells/lane).

To investigate the molecular mechanisms of mTORC1 activation in epithelial cells following MV, we utilized in vitro models of the various forms of VILI (Figure 4, A–C). Airway epithelial cells, like alveolar parenchymal cells, experience injurious physical forces during MV (25). To simulate volutrauma, primary human airway epithelial cells were grown on flexible membranes and subjected to cyclic stretch that models overdistention during injurious MV (26, 27). Volutrauma increased phosphorylation of S6 kinase and ribosomal S6 within 30 minutes in distal small airway epithelial cells (SAECs) and proximal human bronchial epithelial (HBE) cells (Figure 4D and Supplemental Figure 9). mTORC1 activation was dose and time dependent and rapidly decreased following cessation of injurious stretch (Supplemental Figure 9). Injurious stretch also increased phosphorylation of 4E-BP1, a regulator of protein translation that is phosphorylated when mTORC1 is activated (28) (Figure 4D). Volutrauma-induced mTORC1 activation was prevented by allosteric (i.e., rapamycin) and active site (i.e., Torin 2) mTOR inhibitors (Supplemental Figure 9). To model airway collapse and reopening during atelectrauma, we grew SAECs to confluence on collagen-coated glass slides and exposed them to a moving air-liquid interface by propagating a fluid bubble over the monolayer which simulates the movement of edema fluid in ARDS patients undergoing MV (29). As was seen with volutrauma, atelectrauma rapidly increased P-S6 levels in SAECs (Figure 4E). Interestingly, we did not see the increase in S6 kinase or 4E-BP1 phosphorylation that occurred following volutrauma. To model barotrauma, SAECs were cultured at an air-liquid interface and cells were subjected to high levels of transmural pressure (30, 31). Although mTORC1 was activated following barotrauma, the degree of activation was much less than that seen with volutrauma and atelectrauma (Figure 4F). In summary, these data demonstrate that mechanical forces during in vitro volutrauma and atelectrauma activate mTORC1, mirroring the findings from our murine model and patients with ARDS.

### Volutrauma activates mTORC1 through reactive oxygen species-dependent activation of the ERK pathway.

**Figure 5:**
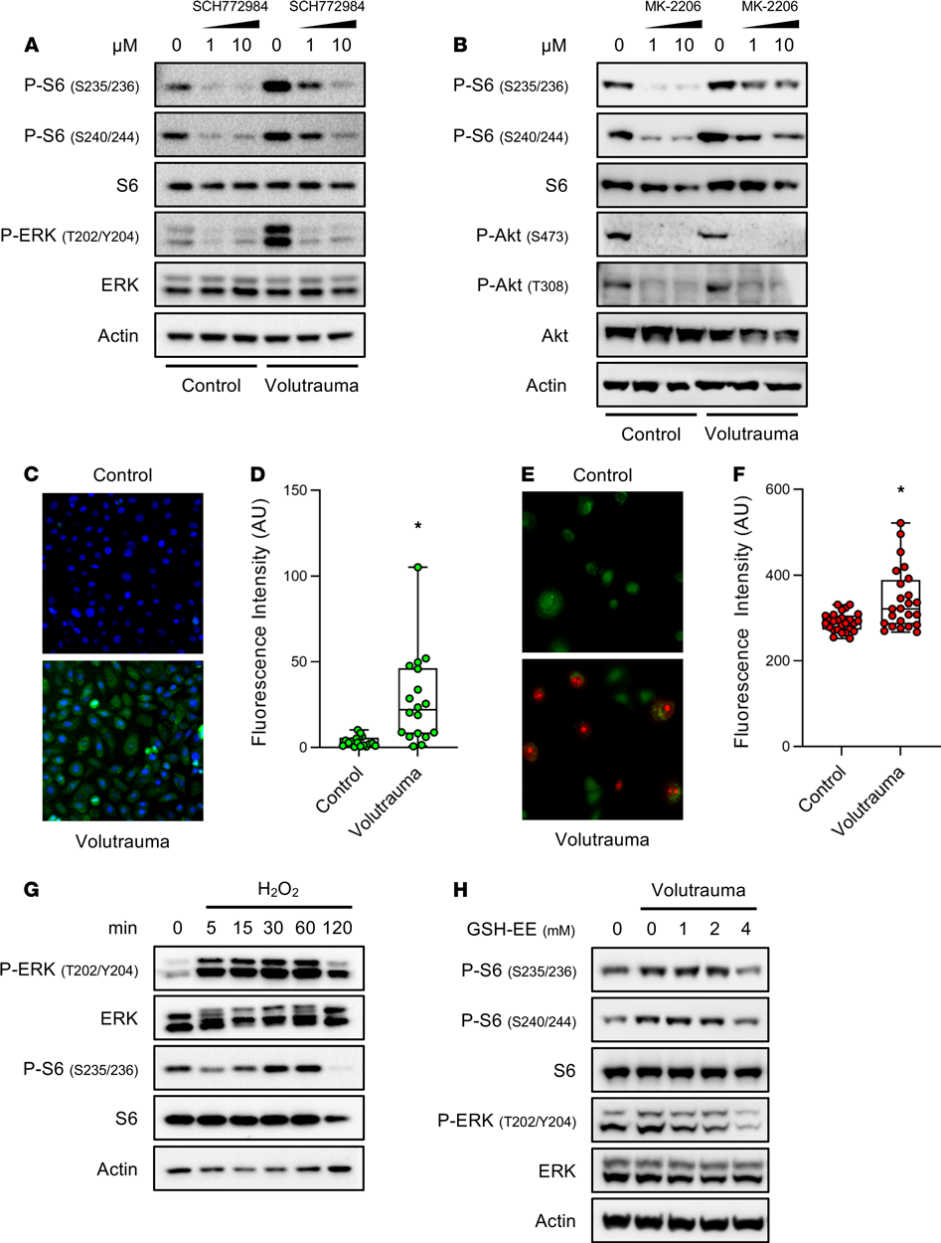
In vitro volutrauma activates mTORC1 through reactive oxygen species-dependent activation of the ERK pathway. (**A**) Human bronchial epithelial cells were subjected to volutrauma (20% equibiaxial stretch, 0.2 Hz, 30 min) in the presence of increasing doses of the ERK 1/2 inhibitor (SCH772984) or vehicle (DMSO) prior to immunoblotting for markers of ERK and mTORC1 activation (pooled protein from *n* = 2 wells/lane). (**B**) SAECs were subjected to volutrauma (24% biaxial stretch, 0.2 Hz, 30 min) in the presence of increasing doses of the AKT inhibitor (MK-2206) or vehicle (DMSO) prior to immunoblotting for markers of AKT and mTORC1 activation (pooled protein from *n* = 2 wells/lane). (**C** and **D**) HBEs were stretched (24% biaxial stretch, 0.2 Hz, 30 min) in the presence of CellRox Green and fluorescence was quantitated in each field (100×). Data log normally distributed, analyzed by Student’s *t* test on log_2_ transformed data (*n* = 18 fields/group). (**E** and **F**) SAECs were stretched (24% biaxial stretch, 0.3 Hz, 30 min) in the presence of mitoSOX (Red) and calcein AM (Green) prior to quantitating the intensity of MitoSOX Red staining in each field (100×). Data not normally distributed, analyzed by Mann-Whitney test (*n* = 27 images per condition). (**G**) Small airway epithelial cells were treated with 500 μM hydrogen peroxide (H_2_O_2_) for increasing amounts of time prior to immunoblotting for markers or ERK and mTORC1 activation (*n* = 1 well/lane). (**H**) SAECs were treated with increasing doses of GSH-EE 30 minutes prior to volutrauma (20% equibiaxial stretch, 0.2 Hz, 30 min) and immunoblotting for markers of mTORC1 and ERK activation (*n* = 2 wells/lane). Box blots show median ± interquartile range and whiskers define min and max values. *P < 0.05 for all panels.

We sought to determine the molecular mechanisms by which injurious ventilation activates mTORC1. Several canonical signaling pathways integrate biochemical and physiologic cues and activate mTORC1 via a series of phosphorylation events, including the ERK and AKT pathways. To determine whether these pathways mediate mTORC1 activation in our volutrauma model, we treated human primary airway epithelial cells with a specific ERK1/2 (SCH772984) or AKT (MK-2206) inhibitor immediately prior to injurious stretch. In vitro volutrauma rapidly increased ERK1/2 phosphorylation that was potently inhibited by SCH772984 (Figure 5A). ERK inhibition completely abrogated the increase in S6 phosphorylation following injurious stretch, indicating that stretch induced mTORC1 activation is ERK dependent (Figure 5A). In contrast to ERK activation by volutrauma, in vitro stretch did not increase AKT phosphorylation (Figure 5B). Treatment with MK-2206 potently inhibited basal AKT and mTORC1 activation, but did not prevent S6 phosphorylation during volutrauma, indicating that VILI-induced mTORC1 activation is AKT independent (Figure 5B). A variety of second messengers can activate ERK1/2 in the lung epithelium, including cellular reactive oxygen species (32, 33). Specifically, hydrogen peroxide (H_2_O_2_) can activate ERK1/2 and mTORC1 in certain cell types (34–36). To determine if cellular ROS are involved in ERK-dependent mTORC1, airway epithelial cells were subjected to volutrauma in vitro and cellular ROS were measured. Total ROS increased following 30 minutes of volutrauma in airway epithelial cells (Figure 5, C and D) that were, in part, from mitochondria (Figure 5, E and F). Treatment of airway epithelial cells with exogenous H_2_O_2_ increased phosphorylation of ERK1/2 within 5 minutes and preceded a dose-dependent activation of mTORC1 that peaked 30–60 minutes after activation (Figure 5G and Supplemental Figure 10). To determine if stretch-induced mTORC1 activation was dependent on the release of cellular ROS, we treated SAECs with glutathione ethyl ester (GSH-EE), a cell permeable form of cellular antioxidant glutathione (37), and found that stretch-induced ERK and mTORC1 activation were abrogated (Figure 5H). These data indicate that the mechanism of mTORC1 activation during volutrauma involves the release of cellular ROS that act as second messengers to activate mTORC1 via the canonical ERK pathway.

### Rapamycin improves lung function during VILI.

**Figure 6:**
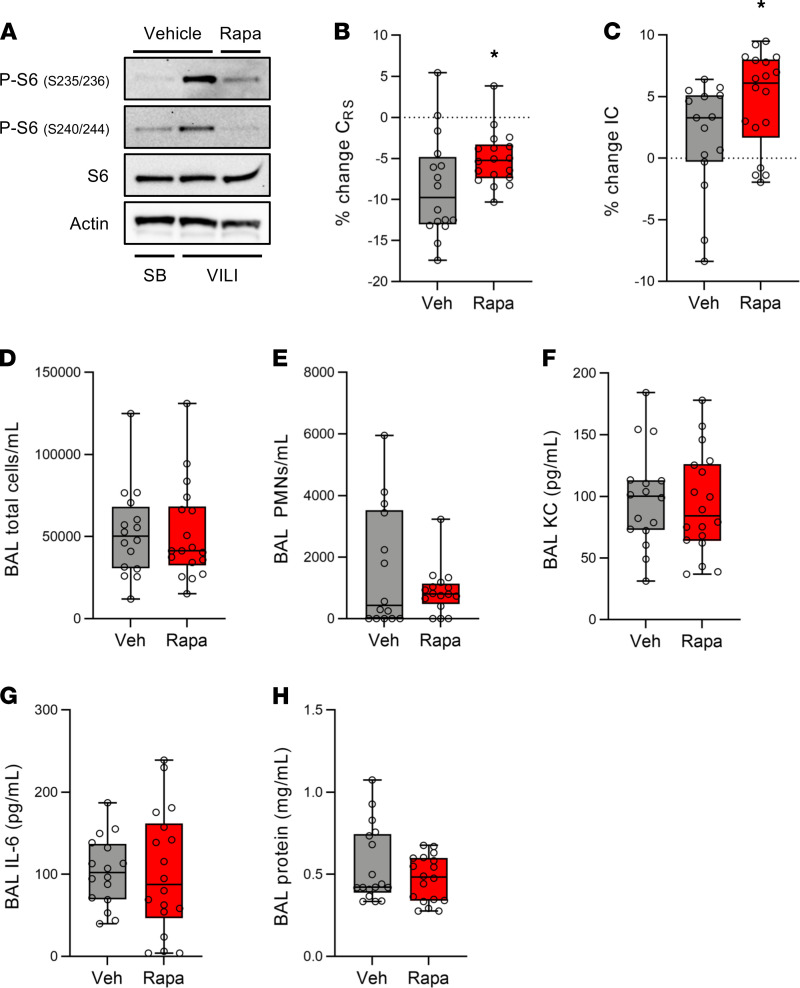
Pharmacologic mTORC1 inhibition attenuates VILI. WT mice were treated with rapamycin (rapa) or vehicle (veh) immediately prior to injurious MV (VILI, TV 12 cc/kg, PEEP 0 cm H_2_O) for 4 hours. (**A**) Following MV, protein was isolated from lung tissue of ventilated (*n* = 6) and SB control (*n* = 4) mice and immunoblotted for phosphorylated S6 (P-S6). (**B**) The percent change in respiratory system compliance (C_RS_) was measured during the 4-hour period of MV. Data normally distributed, analyzed by Student’s *t* test. (**C**) The percent change in inspiratory capacity (IC) was quantitated following 4 hours of VILI. Data not normally distributed, analyzed by Mann-Whitney test. Following mechanical, a bronchoalveolar lavage (BAL) was performed and total inflammatory cells (**D**) and neutrophils (**E)** (PMNs, *n* = 14 veh, *n* = 18 rapa) were measured. KC (**F**) and IL-6 (**G**) levels were measured in BAL fluid by ELISA. BAL protein levels (**H**) were also measured. Box blots show median ± interquartile range and whiskers define min and max values. *P < 0.05, *n* = 16 for veh and *n* = 18 for rapa unless otherwise noted.

To determine the therapeutic potential of inhibiting mTORC1 activation to prevent VILI, we treated WT mice with the mTOR inhibitor rapamycin (5 mg/kg IP) or vehicle control at the time MV was initiated. Mice were subjected to simultaneous volutrauma and atelectrauma (12 cc/kg, PEEP 0 cm H_2_O) for 4 hours. Treatment with a single dose of rapamycin dramatically decreased P-S6 levels in whole lung tissue following VILI (Figure 6A). During injurious ventilation, vehicle-treated mice had decreased respiratory system compliance compared with rapamycin-treated mice (Figure 6B). Over the period of ventilation, rapamycin-treated mice had significant lung recruitment as evidenced by an increase in inspiratory capacity that was not seen in vehicle-treated mice (Figure 6C). Although rapamycin prevented the decrease in lung compliance following VILI, it did not affect recruitment of inflammatory cells (Figure 6, D and E) or alter BAL KC or IL-6 levels (Figure 6, F and G). BAL protein levels were also not different between rapamycin- and vehicle-treated mice (Figure 6H), mirroring our findings in mice with airway epithelial *Tsc2* deletion. These data demonstrate that rapamycin administered at the time MV is initiated decreases mTORC1 activation and prevents impaired lung compliance during MV, independent of lung inflammation or barrier permeability.

### mTORC1 activation exacerbates surfactant dysfunction during injurious ventilation.

**Figure 7:**
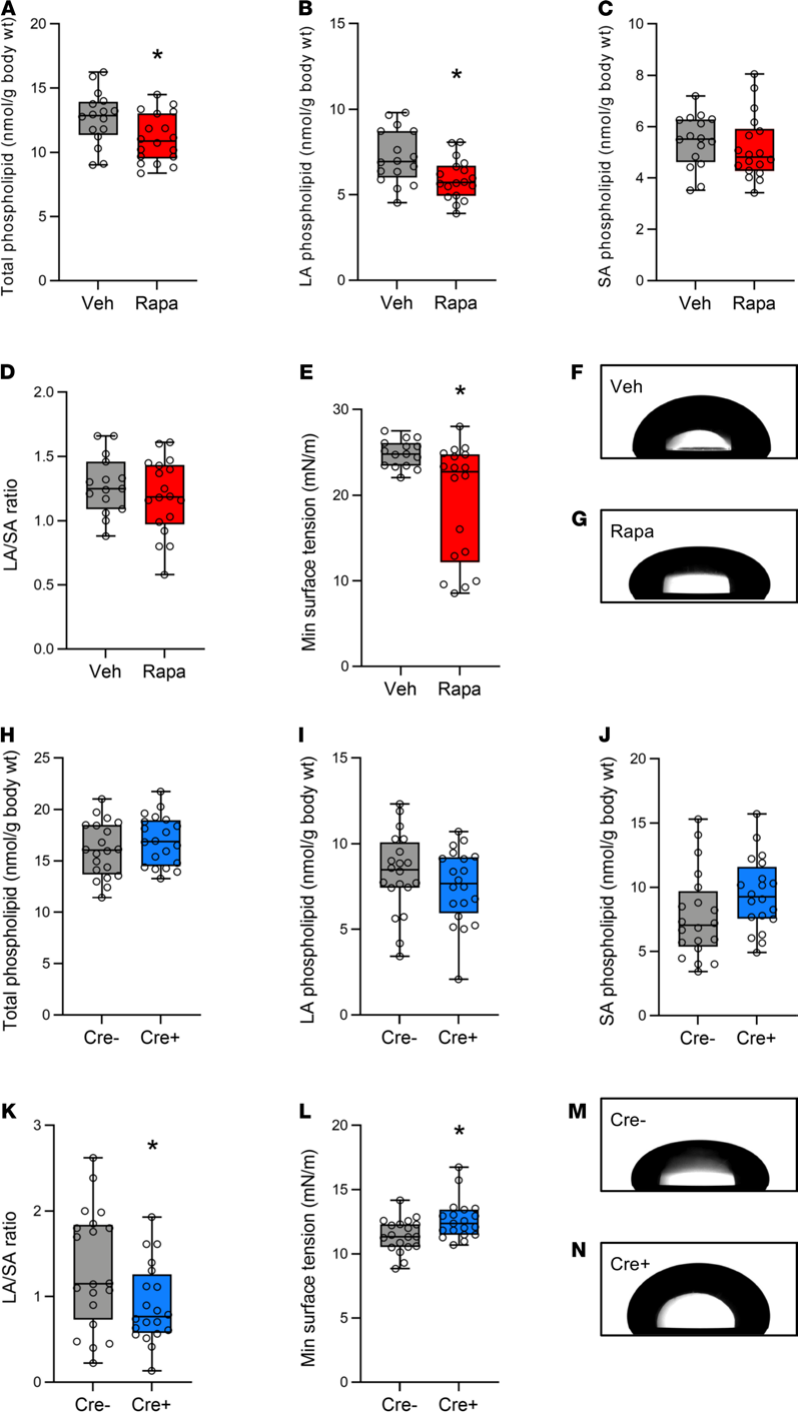
mTORC1 activation exacerbates surfactant dysfunction during injurious ventilation. (**A**) Total phospholipid levels were measured in mice treated with veh or rapa (5 mg/kg) following 4 hours of injurious ventilation (TV 12 cc/kg, PEEP 0 cm H_2_O). Data normally distributed, analyzed by Student’s *t* test. Phospholipid levels were also measured in LA fractions (**B**) and SA fractions (**C**). (**A**–**C**) Data normally distributed, analyzed by Student’s *t* test, *n* = 16 for veh and *n* = 18 for rapa. (**D**) The ratio of LA/SA phospholipid was calculated (*n* = 15 veh, *n* = 18 rapa). (**E**) Minimum surface tension after 15 cycles measured using a CDS in LA fractions from vehicle- and rapamycin-treated mice. Data not normally distributed, analyzed by Mann-Whitney test, *n* = 15 veh, *n* = 18 rapa. (**F** and **G**) Representative images of minimum surface tension from LA fractions of mice treated with rapa or veh prior to VILI. Total (**H**), LA (**I**), and SA (**J**) phospholipid levels were measured in mice with airway epithelial *Tsc2* deletion (Cre+) and Cre– control mice following 4 hours of injurious ventilation (TV 12 cc/kg, PEEP 0 cm H_2_O). The ratio of LA/SA phospholipid was calculated (**K**). Data normally distributed, analyzed by Student’s *t* test, *n* = 20/group for (**H**–**K**). (**L**) Minimum surface tension after 25 cycles measured using CDS in LA fractions from Cre- and Cre+ mice. Data log normally distributed, analyzed by Student’s *t* test on log_2_ transformed data, *n* = 19/group. (**M** and **N**) representative images of minimum surface tension from LA fractions of Cre- and Cre+ mice following VILI. Box blots show median ± interquartile range and whiskers define min and max values. *P < 0.05.

Since our data demonstrate that mTOR inhibition (Figure 6) and *Tsc2* deletion (Figure 2) regulate lung compliance without altering barrier permeability or inflammation, we hypothesized that mTORC1 activation exacerbates lung injury during VILI by impairing surfactant function. Surfactant is a complex mixture of proteins and phospholipids that decreases surface tension and prevents lung collapse under homeostatic conditions. VILI and ARDS cause surfactant dysfunction and impair physiologic lung function by increasing alveolar collapse and decreasing lung compliance (38, 39). To measure the effects of mTOR inhibition on surfactant composition, we separated mouse lung surfactant into the surface-active large aggregate (LA) and inactive small aggregate (SA) fractions and measured phospholipid levels. Mice treated with rapamycin had slightly lower amounts of total and LA phospholipid (Figure 7, A and B). There was no difference in SA phospholipid levels or the ratio of LA/SA phospholipid (Figure 7, C and D). We then fabricated and used a constrained drop surfactometer (CDS) (40, 41) to examine the effects of mTORC1 activation on surfactant function (Supplemental Figure 11). This device allows for the measurement of surface tension dynamics in small volume murine samples, and we validated the CDS by analyzing surface tension versus area plots in serial dilutions of recombinant surfactant (Infrasurf). As shown in Supplemental Figure 11, undiluted Infrasurf exhibited a large hysteresis area and near zero minimum surface tension, while diluted Infrasurf exhibited decreased hysteresis area and increased minimum surface tension (42). After equalizing phospholipid concentrations in LA fractions, rapamycin-treated mice had lower minimum surface tension compared with vehicle-treated mice (Figure 7, E–G). To examine whether mTORC1 activation in airway epithelial cells was regulating surfactant composition and function, we measured phospholipid levels in mice with airway epithelial *Tsc2* deletion and Cre negative controls. There were no significant differences in LA, SA, or total phospholipid levels between Cre- and Cre+ mice, but Cre+ mice did have a decreased ratio of LA/SA phospholipid levels (Figure 7, H–K). Consistent with our findings from rapamycin-treated mice, we found that Cre+ mice (with increased mTORC1 activation) had significantly higher minimum surface tension in the LA fraction (Figure 7, L–N). These data demonstrate an association between mTORC1 activation during VILI and altered surfactant composition and function.

**Figure 8:**
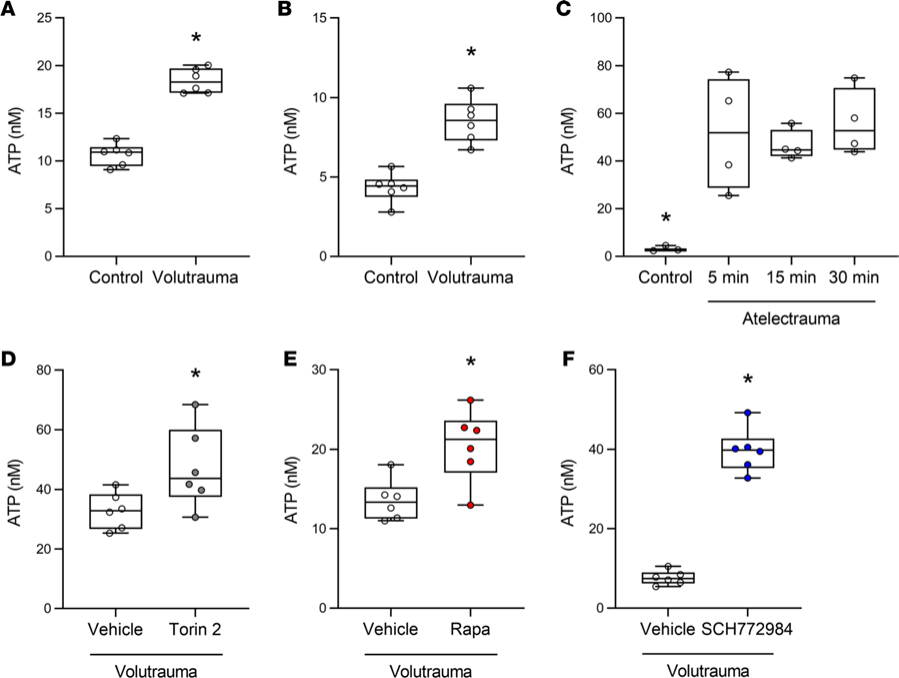
mTORC1 activation impairs release of the surfactant secretagogue extracellular ATP in response to volutrauma. (**A**) SAECs were subjected to volutrauma (24% stretch, 0.2 Hz, 30 min) or static culture (control) prior to measuring extracellular ATP. Data normally distributed, analyzed by Student’s *t* test, *n* = 6 wells/condition. (**B**) HBE cells were subjected to volutrauma (24% stretch, 0.2 Hz, 30 min) or static culture (control) prior to measuring extracellular ATP. Data normally distributed, analyzed by Student’s *t* test, *n* = 6 wells/condition. (**C**) SAECs on polyacrylamide gels were subjected to atelectrauma or control for varying amounts of time prior to measuring extracellular ATP. Data normally distributed, analyzed by 1-way ANOVA with Sidak’s post hoc test, *n* = 4 for all time points except control *n* = 3. (**D**–**F**) HBE cells were treated with Torin 2 (**D**) (10 nM), rapamycin (**E**) (10 nM), SCH772984 (**F**) (10 μM), or appropriate vehicle control 1 hour prior to in vitro volutrauma. ATP concentration was measured in media following 30 minutes of injurious stretch. Data normally distributed, analyzed by Student’s *t* test, *n* = 6 wells/group. Box blots show median ± interquartile range and whiskers define min and max values. *P < 0.05 versus all groups.

*mTORC1 activation impairs release of the surfactant secretagogue extracellular adenosine triphosphate (ATP) in response to volutrauma*. During physiologic respiration, stretch of type I alveolar epithelial cells (AT1) stimulates surfactant release from adjacent type II alveolar epithelial cells (AT2) through the controlled release of ATP into the extracellular space and subsequent binding to purinergic receptors on AT2 cells (43, 44). Stretch-induced release of surfactant plays a key role in surfactant secretion under homeostatic conditions and is an important compensatory response to mitigate lung dysfunction during injurious ventilation (38, 45, 46). We hypothesized that airway epithelial cells also release ATP in response to VILI but that mTORC1 activation in these cells impairs this compensatory response and exacerbates surfactant dysfunction. To test this hypothesis, we subjected SAECs and HBE cells to in vitro volutrauma and found a 2- to 5-fold increase in the concentration of extracellular ATP released into the culture medium (Figure 8, A and B). A similar increase in extracellular ATP was seen when SAECs were subjected to in vitro atelectrauma (Figure 8C). Interestingly, in vitro barotrauma did not increase extracellular ATP levels in either SAECs or HBE cells suggesting that different mechanical forces may have unique effects on the release of extracellular ATP (Supplemental Figure 12). mTORC1 inhibition with rapamycin or Torin 2 significantly increased the release of extracellular ATP following volutrauma (Figure 8, D and E). Given that volutrauma-induced mTORC1 activation is ERK dependent, we treated HBE cells with an ERK inhibitor prior to stretch and found that ERK inhibition prior to volutrauma also increased the release of extracellular ATP (Figure 8F). These data indicate that airway epithelial cells release ATP into the alveolar space following volutrauma and atelectrauma. However, mTORC1 activation in these cells limits the release of extracellular ATP which can be enhanced by treatment with mTORC1 or ERK inhibitors. Paracrine release of ATP from airway epithelial cells may be a compensatory mechanism to increase surfactant release from adjacent AT2 cells during injurious ventilation.

## Discussion

Classically, favorable growth conditions (e.g., presence of nutrients and growth factors) activate mTORC1, and cellular stress (e.g., starvation) inhibits mTORC1 (47, 48). In contrast to canonical mTORC1 activation in the setting of favorable growth conditions, we found that this pathway was activated in lung epithelial cells during injurious MV in preclinical animal models, in vitro models of VILI, and patients with ARDS. Deletion of *Tsc2*, a negative regulator of mTORC1, in airway epithelial cells increased mTORC1 activation and impaired lung function following injurious MV (Figure 2). Conversely, treatment with an mTOR inhibitor (i.e., rapamycin) at the time that MV was initiated prevented the decrease in lung compliance that occurred in vehicle-treated mice (Figure 6). Pharmacologic intervention at the time MV begins is an appealing and practical therapeutic strategy that could be translated to patients with respiratory failure that require ventilatory support.

Despite changes in lung function and oxygenation in our in vivo experiments, neither genetic deletion of *Tsc2* nor pharmacologic mTOR inhibition altered lung inflammation or barrier permeability (Figures 2 and 6). These findings suggest that mTORC1 activation during VILI may impair lung compliance by regulating surfactant function. Surfactant dysfunction is a pathophysiologic hallmark of ARDS that exacerbates lung injury, although the molecular mechanisms driving this dysfunction have not been elucidated (49). We found that mice with airway epithelial *Tsc2* deletion had increased surfactant dysfunction and rapamycin-treated mice had improved surfactant function, resulting in improved lung compliance (Figures 6 and 7). Our in vitro data suggest that activation of mTORC1 in airway epithelial cells during VILI may regulate surfactant function by inhibiting the release of extracellular ATP (Figure 8). Paracrine release of ATP from AT1 cells is known to stimulate surfactant release from adjacent AT2 cells (44, 50), and it is possible that ATP release from distal airway epithelial cells may have a similar effect. Further studies are needed to explore this hypothesis in vivo.

Studies from patients with ARDS have shown that decreased phospholipid content, as well as alterations in the biochemical composition of the lipids that compose surfactant, can contribute to surfactant dysfunction (51). In addition, surfactant can be inactivated in the alveolar spaces by secreted phospholipases (52). Interestingly, increased mTORC1 activation is associated with altered cellular lipid metabolism (53), and prolonged treatment with the allosteric mTOR inhibitor, temosirolimus, increases surfactant lipid levels in mice (54). mTOR activation has been shown to play a role in the development of neonatal lung injury which is characterized by inadequate surfactant production (55). In a mouse model of neonatal respiratory distress syndrome from hyperoxia, rapamycin treatment decreased lung injury (56). Our data demonstrate that rapamycin treatment at the time MV was initiated mitigated VILI-induced surfactant dysfunction (Figure 7). This occurred despite rapamycin-treated mice having decreased phospholipid levels in the surface-active LA fraction. Interestingly, mice with airway epithelial *Tsc2* deletion had a decreased ratio of LA to SA phospholipid that is consistent with a dysfunctional surfactant, but unlike the rapamycin-treated mice, there was no difference in the total amount of phospholipid in each of these fractions. This may be due to the effect of mTORC1 inhibition on multiple lung cell types in the rapamycin-treated mice. These data suggest that the mechanism by which mTOR inhibition mitigates surfactant dysfunction may be by altering phospholipid and protein composition or other physicochemical properties of surfactant. Although trials of exogenous surfactant replacement in adults with established ARDS showed no benefits (57, 58), these trials were complicated by the use of different surfactant formulations and concerns about inadequate dosing. Drugs that enhance the release of surfactant or prevent surfactant dysfunction have not been studied in ARDS. Our data indicate that, in the short term, mTORC1 inhibition may be a useful and feasible therapeutic strategy to mitigate surfactant dysfunction during VILI to prevent further injury.

Activation of mTORC1 has been reported in mouse models of inflammatory lung injury including endotoxin-induced lung injury (16, 59, 60), hyperoxia (56), and bacterial infection (61). Similar to our findings, mTORC1 activation was also increased in alveolar and airway epithelial cells following LPS-induced lung injury (59). In genetic mouse models in which mTOR was deleted from alveolar and airway epithelial cells, cytokine levels were decreased and pulmonary edema was increased following LPS-induced lung injury (59). In contrast with these prior reports, we did not see differences in lung inflammation or barrier permeability with mTOR inhibition in our model of injurious ventilation (Figure 6). This may be due to inherent differences in the mechanism of lung injury, the time points studied, or a combination of these factors. mTORC1 activation has also been examined in inflammatory cells such as neutrophils (16) and Th17 cells (62) following endotoxin induced lung injury. Our data suggest that mTOR inhibition can mitigate lung injury during MV independent of inflammation.

mTOR activation has been shown to play a key role in other forms of lung injury. Pulmonary fibrosis is a condition characterized by the deposition of a stiff extracellular matrix, and mechanical cues from the matrix are known to drive the pathogenesis of lung fibrosis (63). Interestingly, expression of mTOR is increased in epithelial cells of patients with pulmonary fibrosis (64), and Chung et al. showed that rapamycin decreased mortality and fibrosis in a murine model of radiation induced fibrosis (65). In contrast to the injurious role of mTORC1 activation in LPS and fibrosis-induced lung injury, mTORC1 activation appears to play a protective role in the development of cigarette smoke-induced emphysema. Yoshida et al. showed that a stress response protein, RTP801, is induced by cigarette smoke and mediates the development of emphysema, in part, by inhibiting mTORC1 (15). More recently, Houssaini et al. showed that activation of mTORC1 and mTORC2 increased senescence in lung cells from patients with COPD or in mice with hyperactivated mTOR in alveolar epithelial cells (66). These data demonstrate the complex and pleiotropic role of mTOR activation during different mechanisms of lung injury and suggest that a deeper understanding of the pathobiologic mechanisms that lead to lung injury will be important for developing targeted therapies for these patients.

Activation of mTORC1 by mechanical stimuli has been described in skeletal and cardiac muscle in the context of compensatory hypertrophy (12–14, 67) and as a key regulator of bone and cartilage growth during development (68, 69). Canonical activation of mTORC1 by growth factors involves phosphorylation and subsequent inhibition of tuberin, a constitutive inhibitor of mTORC1 encoded by the *Tsc2* gene, by upstream kinases such as ERK and AKT (28). Prior data regarding the role of AKT in mechanical mTORC1 activation in skeletal muscle have been mixed. Bodine et al. used in vivo rat models to demonstrate that AKT was activated following contractile stress in skeletal muscle in a model of compensatory hypertrophy and inhibited in a model of muscle atrophy (14). Using an ex vivo model of skeletal muscle loading, Hornberger et al. showed that tensile stretch activated mTORC1 in an AKT-independent fashion (70). Similarly, our data demonstrate that mechanical activation of mTORC1 in lung epithelial also occurs in an AKT-independent manner (Figure 5).

In contrast to canonical activation of mTORC1, biomechanical activation in muscle can occur in the absence of growth factors (70) via activation of the ERK pathway (13). Similar to force-induced mTORC1 activation in muscle, activation of the complex in lung epithelial cells did not require the presence of exogenous growth factors (Figures 4 and 5). Despite being growth factor-independent, our data demonstrate that injurious stretch in lung epithelial cells rapidly activates the ERK1/2 pathway and that volutrauma-induced mTORC1 activation is ERK-dependent (Figure 5). A variety of second messengers can activate ERK1/2 in lung epithelial cells. Given that physical forces during MV are known to release reactive oxygen species (9, 71) and mTORC1 can be regulated in a redox sensitive fashion (72, 73), we examined the role of ROS in activating mTORC1 during VILI. Our data demonstrate that exogenous ROS (i.e., H_2_O_2_) or volutrauma-induced release of cellular ROS rapidly activate mTORC1 (Figure 5 and Supplemental Figure 10) and that this activation is inhibited by the antioxidant glutathione (Figure 5). This finding is consistent with prior data showing that short-term exposure to ROS can activate mTORC1 in a variety of cell lines (34, 35) and demonstrates this effect for the first time in primary human lung epithelial cells. Excessive production of free radicals in the context of lung injury has been shown to mediate the development of inflammation, barrier permeability, and cell death (71, 74). We believe our data uncover a novel mechanism by which ROS drive the development of lung injury by acting as second messengers that activate mTORC1 signaling.

There are several limitations of our study that are worth noting. Although, pharmacologic mTOR inhibition prevented the decrease in lung compliance during injurious ventilation, it remains to be determined whether this strategy will be efficacious in the setting of preexisting lung injury as occurs in patients with ARDS. Our mouse model to study mTORC1 activation in airway epithelial cells used a constitutively activated promoter to express Cre recombinase and increase mTORC1 activation in CC10 expressing cells during development and in adult mice. Several groups have indirectly implicated a role for mTOR signaling in lung development (75, 76). Our data are consistent with those of Ikeda et al. (55) who generated mice with increased mTORC1 activation in lung epithelial cells during lung development using an epithelial cell specific transgenic mouse expressing a constitutively activated upstream regulator of mTORC1 (i.e., AKT1). They found that when these mice were born at full term they had transient tachypnea and impaired alveolarization at the time of birth, but these abnormalities had improved by postnatal day 2 and they had normal lung histology by the time the mice reached maturity. Although it is possible that mTORC1 hyperactivation via *Tsc2* deletion in CC10 expressing cells led to a similar phenotype in the very early postnatal period, we did not detect any obvious respiratory abnormalities after birth and did not examine lung histology at this very early time point. Clinically, mTOR inhibitors such as rapamycin are used as immunosuppressants after solid organ and stem cell transplantation, and the systemic administration of mTOR inhibitors in the context of pneumonia or other infections that lead to ARDS may be detrimental. In the current study, we focused on mTORC1 activation in airway epithelial cells because this was the most prominent site of mTORC1 activation in lung tissue from mice subjected to VILI (Figure 1 and Supplemental Figure 3). Given the effects of mTORC1 activation on surfactant composition and function (Figure 7), future studies should examine the role of mTORC1 alveolar epithelial cells.

Several mTOR inhibitors are currently FDA-approved for the treatment of tuberous sclerosis complex and to prevent lung function decline in patients with lymphangioleiomyomatosis (77). In our in vivo studies, we administered rapamycin immediately prior to the initiation of MV, which is particularly relevant for preventing lung injury in the context of MV. In summary, our data demonstrate that injurious physical forces during MV activate mTORC1 in lung epithelial cells and that activation of this pathway in this context impairs lung function. Future studies will be needed to further explore the role of mTORC1 inhibition to prevent impaired lung function in ICU patients undergoing MV.

## Methods

### Reagents.

Rapamycin and Torin 2 were purchased from LC Laboratories. SCH772984 and MK-2206 were purchased from SelleckChem. ARL 67156 was purchased from Tocris. Glutathione ethyl ester (buffered to pH 7.4 prior to use), antimycin A, perchloric acid, and ammonium molybdate were purchased from Sigma-Aldrich. Ascorbic acid was purchased from VWR. MitoSOX, CellROX Green, calcein, and propidium iodide were purchased from Invitrogen. Calfactant (Infasurf) was purchased from The Ohio State University Wexner Medical Center pharmacy. A complete list of antibodies can be found in the Supplemental Materials.

### Cell culture.

Primary human SAEC were obtained from Lonza and PromoCell and grown in the manufacturer’s suggested growth medium. Primary HBE cells were also obtained from Lonza and grown in the recommended growth medium. About 12 hours prior to in vitro models of VILI, cells were washed with warm PBS and changed to the corresponding basal medium with penicillin/streptomycin (100 U/mL, 100 μg/mL; Gibco) and amphotericin B (0.25 μg/mL; Gibco) but without growth factors or serum.

### Mouse tracheobronchial epithelial cell isolation.

Tracheobronchial epithelial cells were isolated from *CC10*^Cre^/*Tsc2*^flox/flox^ and *Tsc2*^flox/flox^ mice as previously described (23). Briefly, the trachea and proximal bronchi were isolated and digested in 0.15% pronase (Roche) overnight at 4°C. The digestion was stopped with 10% FBS (Gibco) and cells were pelleted for DNase treatment (Sigma-Aldrich). Following adherence purification, cells in the suspension were collected for analysis by immunoblotting.

### Mouse strains.

Mice expressing homozygously floxed *Tsc2* alleles (*Tsc2*^flox/flox^) and mice expressing Cre recombinase under the control of the CC10 promoter (*CC10*^Cre^) were provided by Elizabeth Henske (Brigham and Women’s Hospital, Boston, MA). *CC10*^Cre^ mice were bred with *Tsc2*^flox/flox^ to generate mice with airway epithelial specific deletion of Tsc2. *mT/mG* reporter mice were obtained from Jackson Laboratory (007576). *CC10*^Cre^ mice were bred to *mT/mG* reporter mice (22) to confirm airway epithelium-specific expression of the Cre recombinase. Genotyping was performed by Transnetyx using real-time PCR for both *Tsc2* and Cre alleles. All mice were backcrossed to a pure C57BL/6 background, which was confirmed by genome scanning performed by Jackson Laboratory. WT C57BL/6 mice (000664) were obtained from Jackson Laboratory. Deletion of *Tsc2* was confirmed by immunostaining lung sections for phosphorylated ribosomal S6 protein and immunoblotting for tuberin and phosphorylated ribosomal S6 with protein lysate from isolated tracheobronchial epithelial cells. All mice were housed in pathogen free conditions in vivariums at Brigham and Women’s Hospital and The Davis Heart and Lung Research Institute. Mice were used for experiments at 6–12 weeks of age and provided food and water ad lib.

### Murine models of VILI.

Mice were sedated with a combination of ketamine (Vedco) and xylazine (Akorn) or pentobarbital (Akorn). For experiments using rapamycin, this was administered by intraperitoneal injection following induction of anesthesia. A metal tracheostomy cannula was placed and mice were warmed using a heating pad to prevent hypothermia and were mechanically ventilated for varying periods of time using a flexiVent (SCIREQ). Oxygen saturations were measured by pulse oximetry using the MouseOx system (Starr Life Sciences) and EKG tracings were monitored using the flexiVent system. Lung physiology parameters were measured at baseline and hourly thereafter using the forced oscillation technique. Measurements of lung elastance and airway resistance were obtained from the constant phase model (78) using the integrated flexiWare software (SCIREQ). Standardized recruitment maneuvers were performed prior to each physiologic measurement to prevent atelectasis and standardize volume history (79). For experiments using the CLP/VILI model, mice were anesthetized and underwent CLP (23 gauge needle, 1 hole, 50% ligation) or sham laparotomy as described previously (80), 24 hours prior to MV. At the end of the MV protocol, animals were sacrificed by anesthetic overdose and a bronchoalveolar lavage (BAL) was performed by instilling 1 mL of sterile PBS twice. The BAL fluid was then centrifuged for 10 minutes at 400 g at 4°C. Pelleted cells were treated with RBC lysis buffer (Alfa Aesar), counted by an automated cell counter (Bio-Rad), and cytospins were performed followed by Diff-Quick staining (Fisher Scientific) to obtain differential cell counts.

### Surfactant phospholipid and function measurements.

Following MV, lungs were lavaged with 1 mL of 0.9% NaCl solution 3 times for a total volume of 3 mL. The BAL fluid was then centrifuged for 10 minutes at 400 g at 4°C. The supernatant was centrifuged at 40000 g for 18 minutes to obtain the SA fraction (supernatant) and the LA fraction (pellet), which was concentrated by resuspension in a small volume of 0.9% NaCl solution. Phospholipids were extracted from fractionated pulmonary surfactant samples by the Bligh and Dyer method (81). Phosphorus content was measured by colorimetry following perchloric acid digestion and treatment with ammonium molybdate and ascorbic acid (82). Surfactant surface tension was measured using a CDS based on a prior design (40) that consisted of a modified goniometer, digital camera (ramé-hart), and custom-built pedestal. The CDS was manufactured with 316 stainless steel (Columbus Machine Works) and consists of a circular pedestal with a sharp 60° edge to prevent film leakage. Thirty-five μL droplets of resuspended LA fractions were placed on the pedestal of the CDS. The droplet was oscillated using a programmable syringe pump at a rate of 6 seconds per cycle and compressed to approximately 50% of the initial surface area. This was repeated for multiple cycles per LA sample. A custom-written MATLAB code was used to analyze the droplet shape in each frame to obtain surface tension and surface area measurements. First, the Sobel method was used to detect the droplet edge in each frame. Next, the differential equations governing force balances for an arbitrary axisymmetric droplet shape (83) were integrated to determine a theoretical droplet shape based on the surface tension, tip radius of curvature, and angle of inclination parameters. Finally, a nonlinear least-squared regression algorithm was used to vary the surface tension and tip radius of curvature parameters until the theoretical droplet shape matched the measured droplet shape. The droplet shape was also integrated to determine the surface area in each frame. This algorithm results in surface tension versus time and surface tension versus area hysteresis loops that were further analyzed for minimum surface tension.

### In vitro volutrauma model.

Cells were cultured on pronectin-coated BioFlex culture plates (Flexcell International Corporation) and grown to confluence. Cells were subjected to varying degrees of biaxial cyclic stretch delivered via a Flexcell Strain Unit FX-5000 for varying amounts of time prior to protein isolation or imaging. Control cells were placed in the stretching device, but not subjected to mechanical stretch.

### In vitro atelectrauma model.

An in vitro model of atelectrauma was used as previously described (29, 84). Briefly, polyacrylamide gels containing 10% w/w acrylamide (Bio-Rad) and 0.25% w/w bis-acrylamide (Bio-Rad) were fabricated on 40 mm glass slides (20 kPa, 200 μm thickness). Polyacrylamide gels or glass slides were coated with collagen type I (Sigma-Aldrich) at a concentration of 5 μg/cm^2^ following surface activation of the glass slide with sulfo-SANPAH (Thermo Fisher Scientific). Cells were cultured on the gels or glass slides and placed in a microfluidic chamber (Bioptechs) with a rectangular silicone gasket to create the channel for cyclic propagation of media at a linear velocity of 30 mm/s for varying amounts of time.

### In vitro barotrauma model.

Oscillatory pressure was applied as described previously (85). Briefly, cells were cultured on Transwell inserts (0.4 μm pore size, PET; Corning) and grown to confluence. Media was removed from the apical chamber of the Transwell and a rubber stopper was inserted into the culture well in order to hermetically seal the apical chamber. Tubing was threaded through the rubber stopper and connected to a manometer and small animal ventilator (Harvard Apparatus). The ventilator was set to 0.2 Hz at a magnitude of 30 cm of H_2_O of oscillatory pressure.

### Extracellular ATP measurements.

Cells were subjected to in vitro models of VILI in the presence of an ectonucleotidase inhibitor ARL 67156 (50 μM) to prevent ATP degradation. Media was collected following in vitro models of VILI and extracellular ATP was measured using a luciferase-luciferin assay (Invitrogen).

### Measurements of cellular reactive oxygen species.

Cells were grown to confluence and changed to basal medium 24 hours prior to in vitro volutrauma. MitoSOX (5 μM) and calcein (4 μM) were added to each well 30 minutes prior to injury. Antimycin A (20 μM) was used as a positive control. Following stretch cells were washed 3 times with PBS, membranes were cut out of the Flexcell plates and inverted into a 60 mm dish containing a small amount of basal medium immediately prior to obtaining fluorescence images. For MitoSOX studies, 9 separate 100X images were captured from each membrane. Cells were defined in ImageJ using an ROI detection algorithm on the calcein-stained images and MitoSOX intensity was quantitated in ImageJ for each cell within capture. Average intensity per cell was calculated for each capture. For CellROX experiments, average fluorescence per high power field was quantitated using ImageJ.

### Immunoblotting.

Cells or lung tissue were lysed in RIPA buffer (VWR) supplemented with protease (Roche Diagnostics) and phosphatase inhibitors (Sigma-Aldrich). Cells were mechanically disrupted using a rubber scraper followed by a freeze/thaw cycle. Lung tissue was disrupted using a tissue homogenizer. Protein extracts were centrifuged to pellet insoluble debris, protein concentration was measured by BCA assay (Thermo Fisher Scientific), and protein concentrations were equalized prior to boiling in the recommended sample buffers supplemented with 2.5% beta-mercaptoethanol (Bio-Rad). SDS-PAGE electrophoresis was performed using 4–12% gradient bis-tris gels or 16% tricine gels (Invitrogen), and proteins were transferred to nitrocellulose membranes (Bio-Rad). Membranes were incubated in primary antibodies overnight at 4°C following blocking in 5% w/v nonfat dry milk in tris-buffered saline with 0.1% Tween 20 (TBST). After incubation with HRP-conjugated secondary antibodies, proteins were visualized using SignalFire Elite ECL Reagent (Cell Signaling Technology) and imaged using a ChemiDoc XRS+ System (Bio-Rad). Quantitative assessment was performed using Image Lab software (Bio-Rad).

### Immunohistochemistry and image analysis.

Mouse lungs were perfused free of blood with 10 mL of sterile saline and inflated with 10% formalin to a pressure of 30 cm H_2_O prior to overnight fixation. Following fixation, lung tissue was embedded in paraffin. For immunostaining, lung sections from mice and humans were deparaffinized and rehydrated prior to boiling in antigen retrieval buffer (10 mM Na citrate/0.5% Tween 20) for 30 minutes. Tissue was permeabilized with 0.3% Triton X, and quenching and blocking were performed using a cell and tissue staining kit (R&D Systems) according to the manufacturer’s protocol. Tissue was incubated with anti–phospho-S6 antibody (Ser235/236) in 2% goat serum in PBS overnight at 4°C. Nonimmune serum and no primary antibody controls were performed with each experiment. H&E staining was performed using standard techniques. Blinded review was performed by a veterinary pathologist and a lung injury score was calculated (86). Stained sections were scanned at The Ohio State Wexner Medical Center Digital Imaging Core using a NanoZoomer 2.0-HT Scan system (Hamamatsu Photonics) to generate digital whole-slide images. Quantification of the digital images was performed using the VIS software suite (Visiopharm). A single ROI was then made using a tissue detection application to measure a mean DAB stain intensity of all tissue including within each tissue section. Nontissue areas within tissue sections such as airway and vascular lumens were excluded from the mean intensity measurement.

### Immunofluorescence staining.

Following euthanasia, the lungs were perfused via the right ventricle with 1x Ca^2+^/Mg^2+^-free Dulbecco’s PBS (Gibco) followed by 2% (v/v) paraformaldehyde (PFA) (Thermo Fisher Scientific) in PBS. Lungs were inflated to 20 cm H_2_O and then immersed in 2% PFA for 2 hours. After fixation, lung tissues were embedded in OCT compound (Fisher Scientific) and frozen in 2-methylbutane (Honeywell) in a beaker submerged in liquid nitrogen. OCT embedded lungs were cryosectioned in the coronal plane at 5 μm using a cryostat microtome (Leica CM 1510S). The tissue sections were then boiled in antigen retrieval buffer (10 mM sodium citrate, 0.05% Tween 20, pH 6.0) (Sigma) for 30 minutes before permeabilization in 0.3% (v/v) Triton X-100 (Sigma) in PBS for 5 minutes and blocking in 5% goat serum in PBS (Sigma) for 1 hour at room temperature. The sections were immunostained for CC10 using unconjugated rabbit anti-uteroglobin antibody (Abcam, ab40873) at a 1:400 dilution overnight at 4°C followed by incubation of goat anti-rabbit Alexa Fluor 488 secondary antibody (Invitrogen, A-11034) at a 1:200 dilution at room temperature for 1 hour. Prior to sequential staining with another antibody, the sections were incubated in normal rabbit IgG (Cell Signaling Technology, 2729) at a 1:250 dilution for 1 hour at room temperature. The sections were sequentially stained with rabbit anti–Phospho-S6 ribosomal protein (Ser240/244) antibody conjugated to Alexa Fluor 594 (Cell Signaling Technology, 9468) at a 1:50 dilution overnight at 4°C. All antibodies were diluted in 1% BSA (Fisher Scientific) in PBS/0.3% (v/v) Triton X-100. The sections were then stained with Hoescht 33258 at a 1:10,000 dilution at room temperature for 5 minutes and mounted with ProLong Gold Antifade (Thermo Fisher Scientific). Images were acquired with a fluorescence microscope (Olympus IX81-ZDC) and processed using Fiji software.

### BAL protein and cytokine analysis.

Protein concentrations were measured in BAL fluid using a standard BCA assay (Thermo Fisher Scientific) and various analytes, including IL-6, KC, and VEGF-A, which were measured by ELISA (R&D Systems) according to the manufacturer’s instructions.

### Study approval.

All animal studies were approved by the Institutional Animal Care and Use Committee at The Ohio State University (2013A00000105-R2) and performed in accordance with NIH guidelines. Animals were randomly assigned to treatment groups and used at 6–12 weeks of age unless otherwise noted. Lung tissue was obtained from the Brigham and Women’s Department of Pathology and clinical data were collected from subjects under an approved IRB protocol 2015P002273.

### Statistics.

Data were analyzed using GraphPad Prism versions 7 and 8. Data are represented as box and whisker plots (median ± min/max) unless otherwise noted. For in vivo studies, sample size was determined by performing a pilot experiment and the sample mean and standard deviation estimates were used to calculate the final sample size with power of 0.8 and α = 0.05. Normality and log-normality were tested using the Shapiro-Wilk test. For comparisons between 2 groups, a Student’s 2-tailed *t* test and Mann-Whitney *U* test were used for parametric and nonparametric data respectively. For comparisons between multiple groups, a 1-way or 2-way ANOVA was performed depending upon the number of experimental groups. If a significant effect was determined by ANOVA, a Sidak post hoc test (for 1-way ANOVA) or a Holms-Sidak post hoc test (for 2-way ANOVA) was performed for individual group comparisons. A *P* value *<* 0.05 was considered statistically significant. Outliers were identified using nonlinear regression via the ROUT method with a Q threshold of 1% (87). For clinical data, association analyses between pairs of variables were conducted with Fisher’s exact tests (for categorical variables) and 2-tailed *t* tests or Kruskal-Wallis tests (for continuous variables as appropriate based on the normality of the data) using SAS version 9.4.

## Author contributions

JAE and RMB conceived and designed the study. JAE, HL, QF, AS, WZ, CI, CMB, PP, AAR, MPV, RKP, DPAM, DBB, AHM, HCL, NHC, EMS, SNG, JWC, RDH, and LMS acquired, analyzed, and interpreted data. JAE and HL drafted the manuscript. JAE, HL, RMB, JWC, SNG, EPH, and RDH critically revised the manuscript for intellectual content. All authors approved the final version of the manuscript and agree to be accountable for all aspects of the study.

## Supplementary Material

Supplemental data

## Version 1 06/17/2021

In-Press Preview

## Version 2 07/22/2021

Electronic publication
