# Supplementary material for: mTORC1 is a mechanosensor that regulates surfactant function and lung compliance during ventilator-induced lung injury
Source: JCI Insight. 2021 Jul 22;6(14):e137708. doi: 10.1172/jci.insight.137708 (PMC8410036; doi:10.1172/jci.insight.137708)
Supplement: Supplemental data [file jciinsight-6-137708-s180.pdf]

Supplemental Materials

List of antibodies

NAME	COMPANY	PRODUCT #
Phospho-S6 Ribosomal Protein (Ser235/236)	Cell Signaling Technology	2211
Phospho-S6 Ribosomal Protein (Ser240/244)	Cell Signaling Technology	2215
Phospho-S6 Ribosomal Protein (Ser240/244) (D68F8) XP® Rabbit mAb (Alexa Fluor® 594 Conjugate)	Cell Signaling Technology	9468
S6 Ribosomal Protein	Cell Signaling Technology	2217
Tuberin/TSC2	Cell Signaling Technology	4308
Phospho-p44/42 MAPK (Erk1/2) (Thr202/Tyr204)	Cell Signaling Technology	4370
p44/42 MAPK (Erk1/2)	Cell Signaling Technology	4695
Phospho-p70 S6 Kinase (Thr389)	Cell Signaling Technology	9205
p70 S6 Kinase	Cell Signaling Technology	9202
Phospho-4E-BP1 (Thr37/46)	Cell Signaling Technology	2855
4E-BP1	Cell Signaling Technology	9452
Phospho-Akt (Thr308)	Cell Signaling Technology	13038
Phospho-Akt2 (Ser474)	Cell Signaling Technology	8599
Akt (pan)	Cell Signaling Technology	4691
β-Actin	Cell Signaling Technology	3700
Anti-Uteroglobin antibody	Abcam	ab40873
Anti-mouse IgG, HRP-linked	Cell Signaling Technology	7076
Anti-rabbit IgG, HRP-linked	Cell Signaling Technology	7074
Goat anti-Rabbit IgG (H+L) Highly Cross-Adsorbed Secondary Antibody, Alexa Fluor 488	Thermo Fisher Scientific	A-11034

Tables

Supplemental Table 1. Baseline characteristics of mechanically ventilated patients from pathology tissue bank.

	Normal (N=5)	DAD (N=5)	P-value
Age (mean ± SD)	56 ± 11	62 ± 18	0.6
Sex (no. female, n, %)	1 (20)	1 (20)	1.0
Race (no. white, n, %)	5 (100)	4 (80)	1.0
Ever Smoker	3 (60)	2 (67)*	1.0
Pack Years Smoking (median, IQR)	5 (0, 40)	16 (0, 17)*	1.0
Heart Disease (no., %)	0	3 (60)	0.17
Lung Disease (no., %)	1 (20)	1 (20)	1.0
CKD (no., %)	0	3 (60)	0.17
Liver Disease (no., %)	0	1 (20)	1.0
Malignancy (no., %)	2 (40)	1 (20)	1.0
Diabetes (no., %)	0	3 (60)	0.17
Immune Suppression (no., %)	0	1 (20)	1.0

*No smoking data for 2 of the subjects

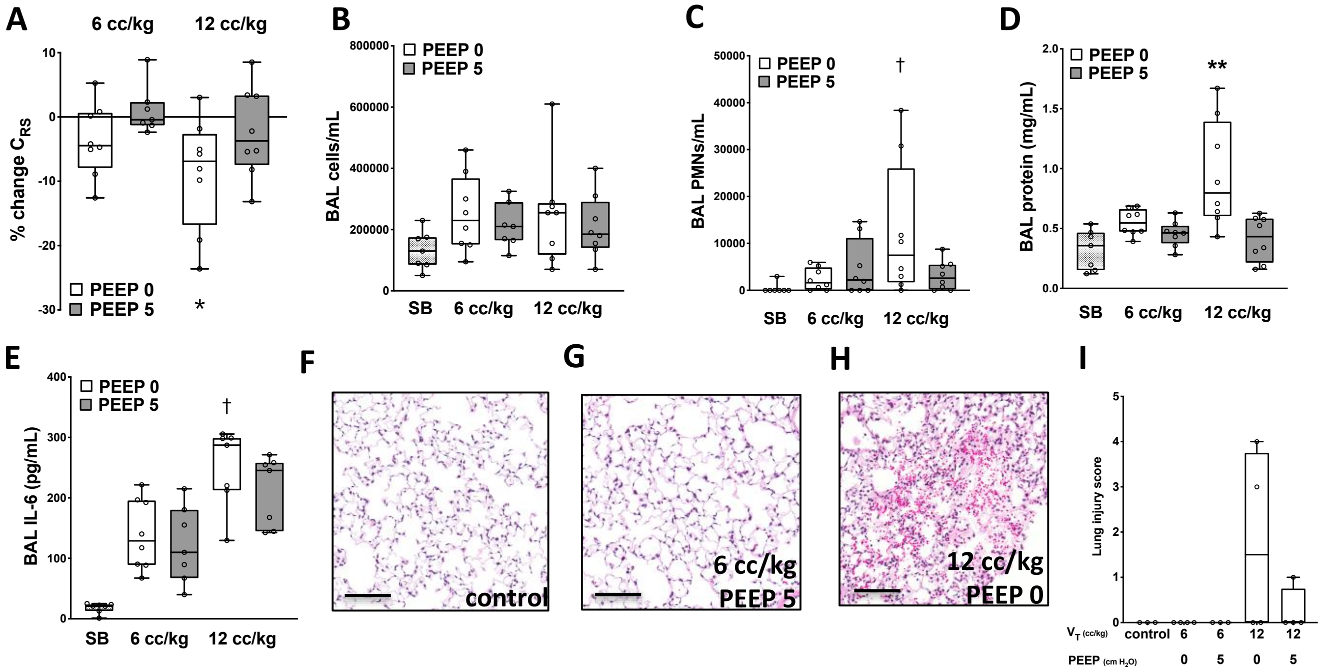

Supplemental Figure 1. Volutrauma and atelectrauma induce lung injury in mice. Wild type mice (n=8/group) were mechanically ventilated with high (12 cc/kg) tidal volume (V_T) to induce volutrauma or low V_T (6 cc/kg) with or without positive end expiratory pressure (PEEP) for 4 hours. A) Mice subjected to simultaneous volutrauma (V_T 12 cc/kg) and atelectrauma (PEEP 0 cm H_2O) had significantly decreased respiratory system compliance (C_{RS}). B-C) Total BAL cell counts were not different between groups but there were significantly more BAL neutrophils (PMNs) in mice ventilated with high V_T without PEEP compared to spontaneously breathing (SB) control mice (n=7). Mice subjected to combined volutrauma and atelectrauma also had a significant increase in BAL protein (D) and interleukin-6 (E) levels. H&E images of lung tissue from SB control mice (F), mice ventilated with low V_T (6 cc/kg) and PEEP (G), and mice subjected to simultaneous volutrauma and atelectrauma (H). I) Lung injury score was assessed by a blinded veterinary pathologist using H&E stained images (black bar=100 μm). *$p<0.05$ vs 12 cc/kg, PEEP 5 by 1-way ANOVA with Tukey's post-hoc test; †$p<0.05$ vs SB controls by 1-way ANOVA with Tukey's post-hoc test; **$p<0.05$ vs all groups by 1-way ANOVA with Tukey's post-hoc test. Box blots show median +/- interquartile range and whiskers define min and max values.

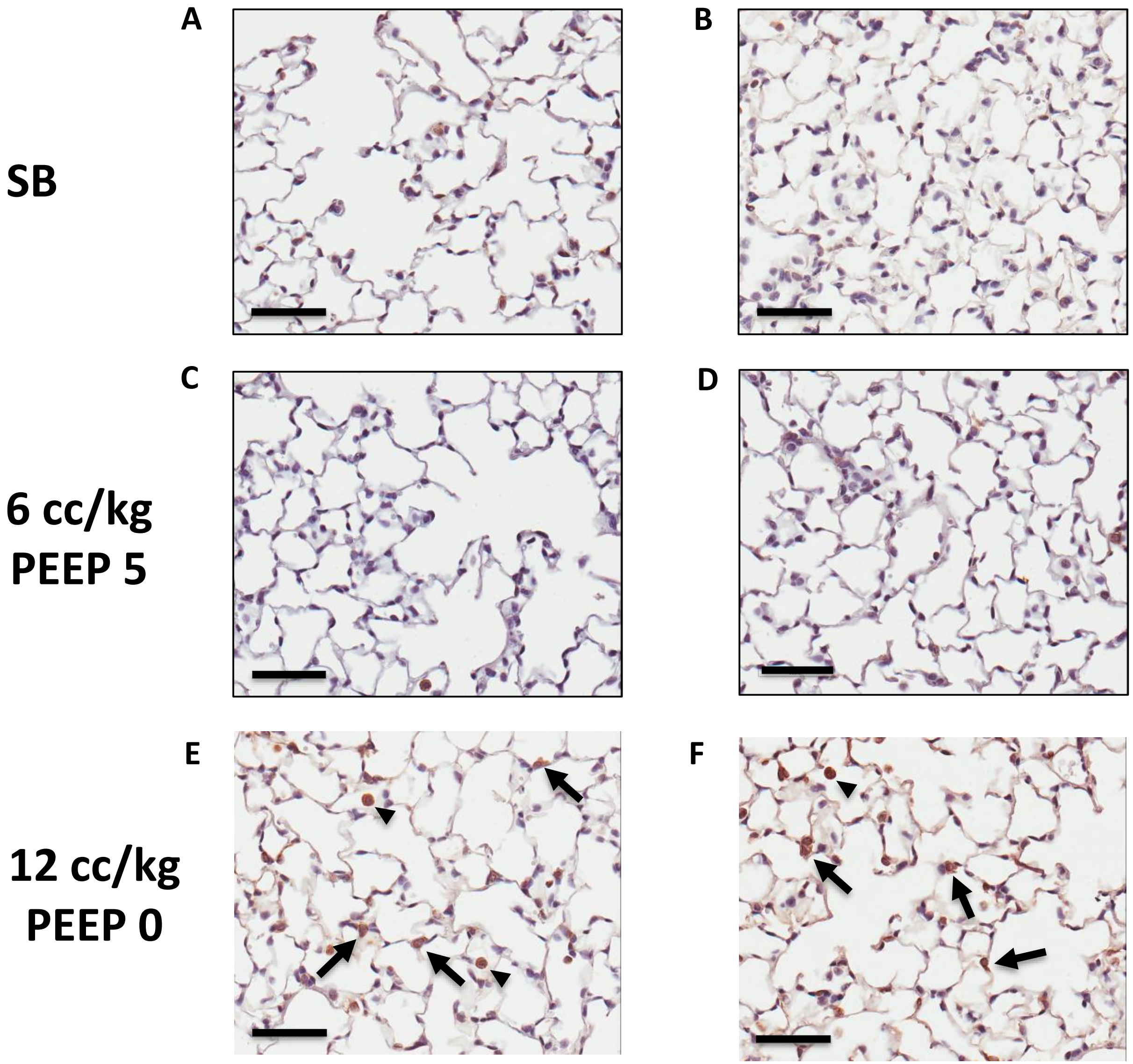

Supplemental Figure 2. Injurious mechanical ventilation activates mTORC1 in lung epithelial cells. Representative alveolar images from lung tissue that was immunostained for P-S6 (Ser235/236) from spontaneously breathing control mice (SB, A-B), mice ventilated with low tidal volume (6 cc/kg) and PEEP (C-D), and mice subjected to simultaneous volutrauma and atelectrauma (E-F) mice. Arrows indicate positively stained cells with type II alveolar epithelial cell morphology and arrowheads indicate positively stained alveolar macrophages. Scale bars=50 μm.

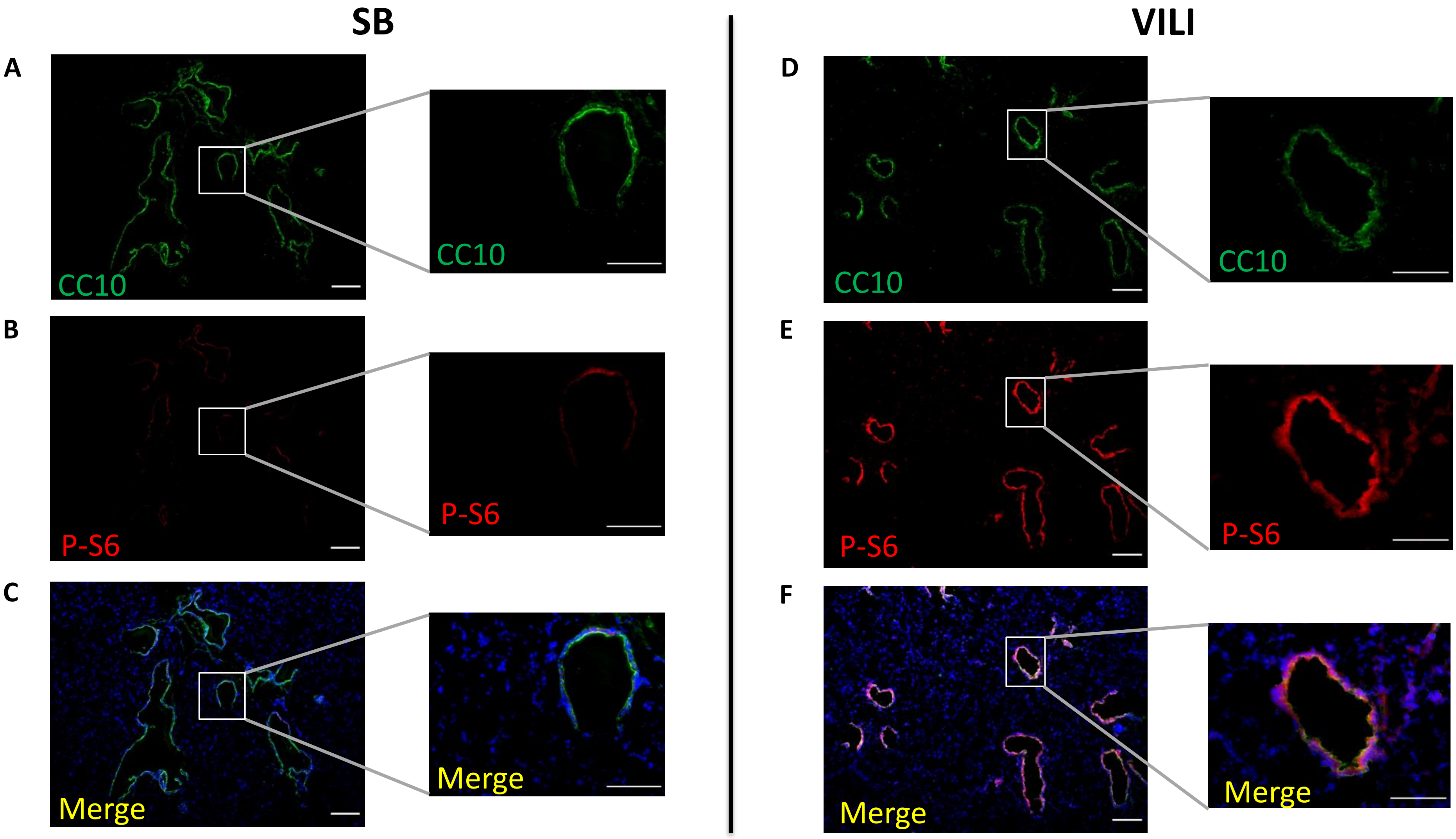

Supplemental Figure 3. Injurious ventilation increases P-S6 levels in CC10 expressing airway epithelial cells. Representative fluorescence images from lung tissue from spontaneously breathing (SB) mice and mice subjected to VILI (12 cc/kg, PEEP 0). Lung tissue was stained for CC10 (green, panels A & D), P-S6 (red, panels B & E). Scale bar in low power images=200μm. Scale bar in high power images=100μm.

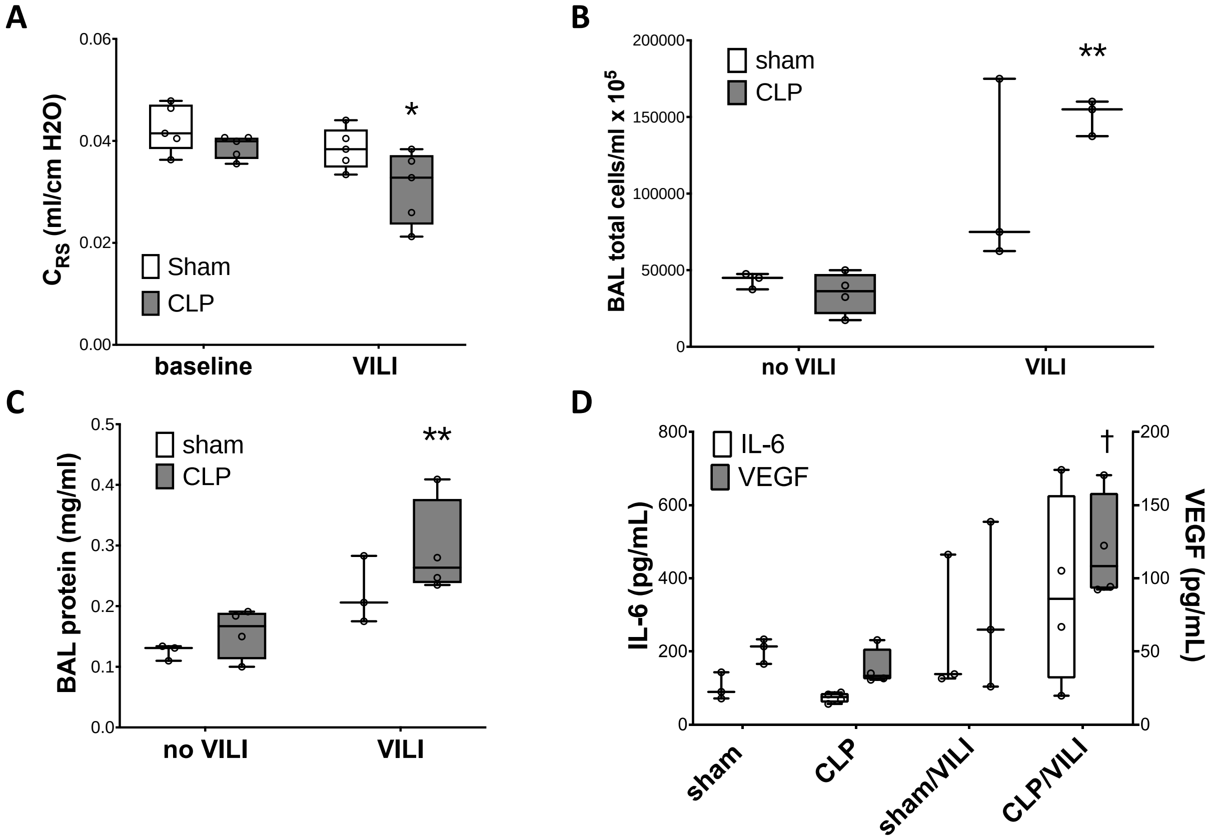

Supplemental Figure 4. Injurious mechanical ventilation following polymicrobial sepsis exacerbates lung injury. Wild type mice were subjected to cecal ligation and puncture (CLP, 23 gauge, 1 hole, 50% ligation) or a sham laparotomy. Mice were allowed to recover for 24 hours and were re-anesthetized and subjected to injurious mechanical ventilation (VILI-V_T 12 cc/kg, PEEP 2.5 cm H_2O) for 4 hours. A) Lung compliance was measured at the time mechanical ventilation was initiated (baseline) and hourly after that. CLP/VILI mice had significantly decreased lung compliance at 4 hours compared to sham/VILI mice. (n=5/group, *p<0.05 vs sham/VILI by 2-way ANOVA with repeated measures and Bonferroni post-hoc test). The combination of CLP and VILI (n=4) also significantly increased the number of BAL inflammatory cells (B), BAL total protein concentration (C), and BAL VEGF levels compared to sham/VILI (n=3) mice. **p<0.05 vs sham & CLP by 2-way ANOVA with Bonferroni post hoc test. †p<0.05 vs CLP by 2-way ANOVA with Bonferroni post hoc test. Box blots show median +/- interquartile range and whiskers define min and max values.

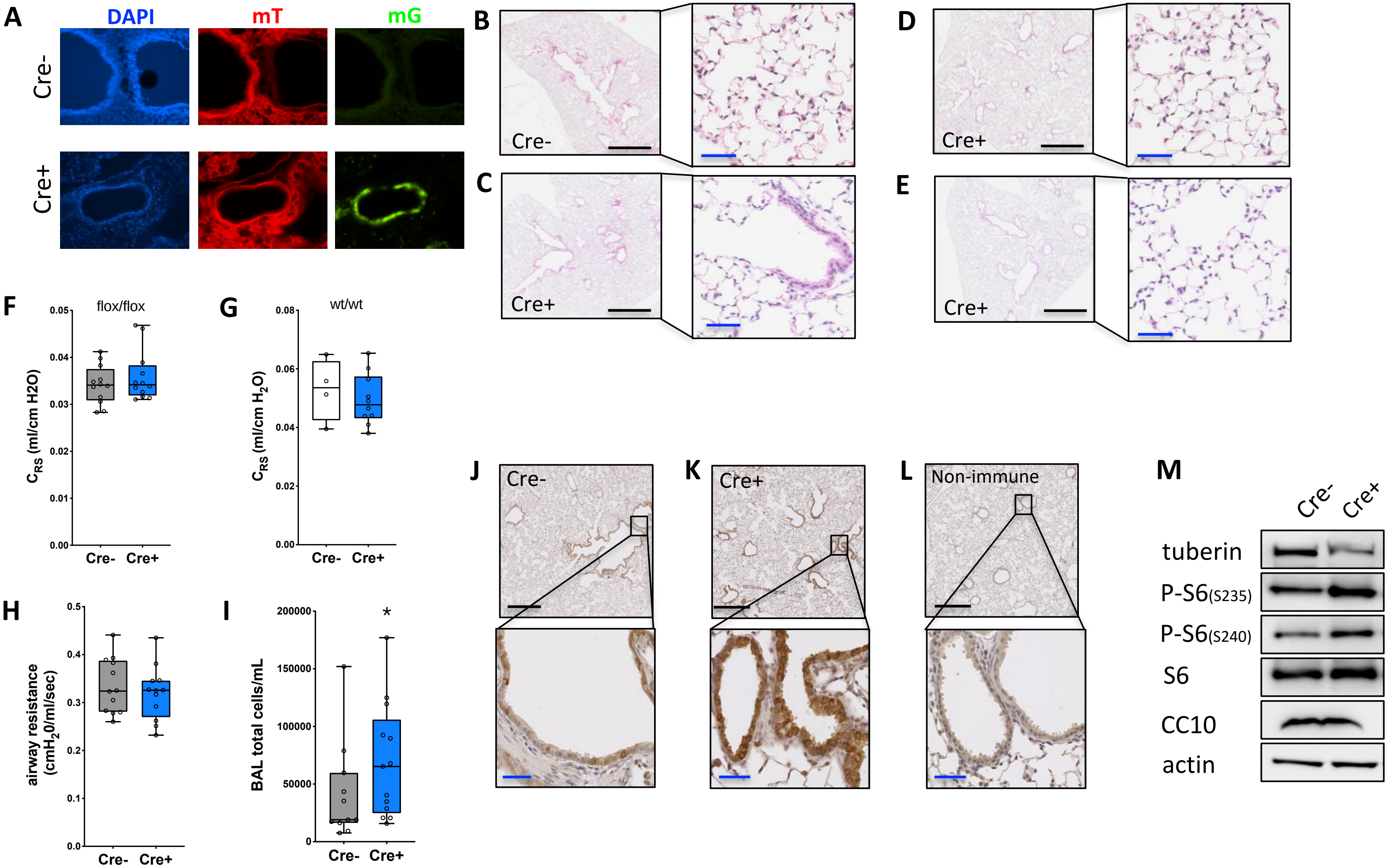

Supplemental Figure 5. Airway epithelial *Tsc2* deletion increases mTORC1 activation but does not alter lung morphology or function in uninjured mice. A) Immunofluorescence images of lung tissue from mT/mG transgenic mice bred to mice expressing Cre recombinase under the control of the CC10 promoter (Cre+) or control mice without Cre recombinase (Cre-). B-E) Representative photomicrographs of H&E stained lung tissue from spontaneously breathing mice with airway epithelial *Tsc2* deletion (Cre+) or controls (Cre-). (black line 1 mm, blue line 50 μm) F) Lung compliance (C_{RS}) from mice with airway epithelial *Tsc2* deletion (Cre+, n=12) or controls (Cre-, n=12). G) Lung compliance (C_{RS}) from mice expressing wild type (WT) *Tsc2* with (Cre+, n=10) or without Cre recombinase (Cre-, n=4) under the control of the CC10 promoter. H) Airway resistance measurements in mice with airway epithelial *Tsc2* deletion (Cre+, n=12) and controls (Cre-, n=12). I) BAL cells from mice with airway epithelial *Tsc2* deletion (Cre+, n=13) and Cre- controls (n=10) Data normally distributed, analyzed by student's t-test. J-L) Representative photomicrographs of lung sections from spontaneously breathing Cre- and Cre+ mice stained for phosphorylated ribosomal S6 (P-S6, Ser235/236) or non-immune rabbit serum (H, black line 500μm, blue line 50μm) M) Immunoblots from isolated tracheobronchial epithelial cells from spontaneously breathing Cre- and Cre+ mice for tuberin (encoded by *Tsc2* gene), markers of mTORC1 activation, and CC10. (pooled protein from n=2 mice/lane). Box blots show median +/- interquartile range and whiskers define min and max values. *$p<0.05$

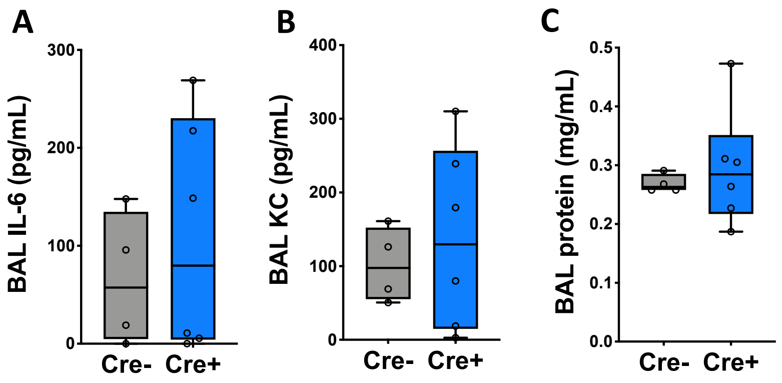

Supplemental Figure 6. BAL IL6, KC, and protein levels are not different in spontaneously breathing mice with airway epithelial *Tsc2* deletion. BAL IL-6 (A), KC (B), and total protein levels from spontaneously breathing Cre- and Cre+ mice. (n=4 for Cre-, n=6 for Cre+). Box blots show median +/- interquartile range and whiskers define min and max values.

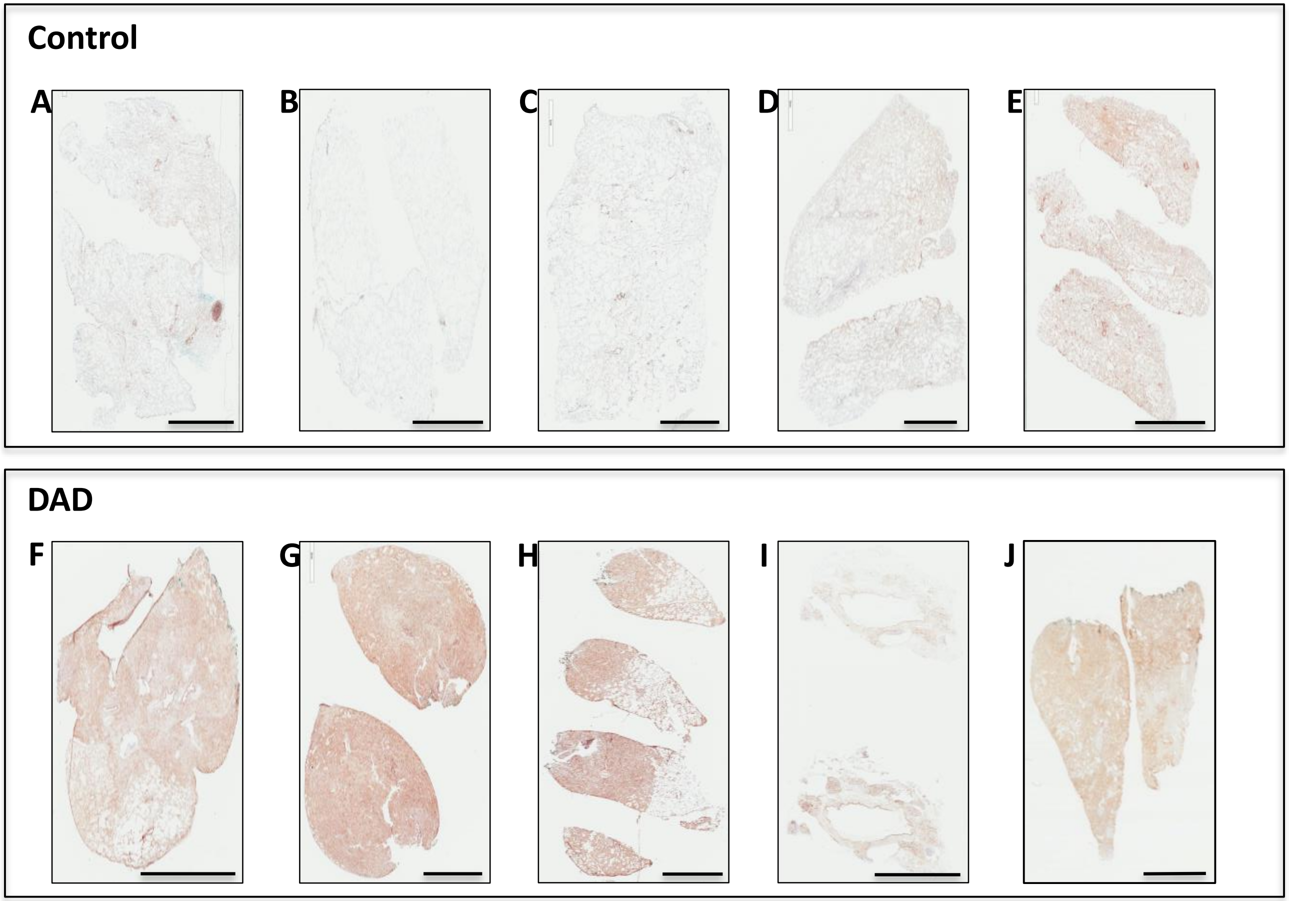

Supplemental Figure 7. mTORC1 is activated in lung tissue from mechanically ventilated patients with diffuse alveolar damage. Whole slide Images of P-S6 (Ser235/236) stained lung tissue from control subjects (A-E) and from patients with diffuse alveolar damage (DAD, F-J). Black bar=5 mm.

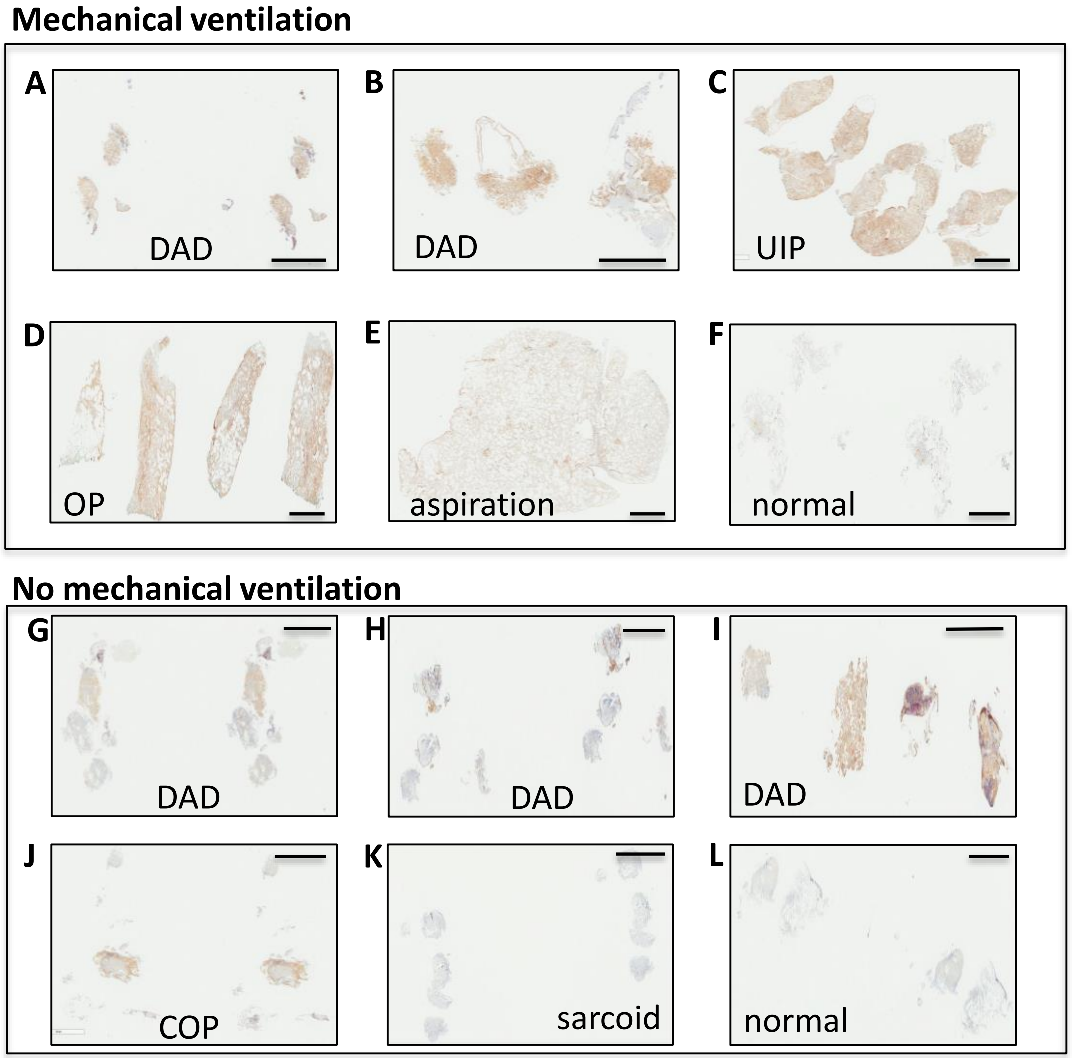

Supplemental Figure 8. Mechanically ventilated patients have increased mTORC1 activation in lung tissue compared to spontaneously breathing patients. Low power images of lung tissue stained for P-S6 (Ser235/236) from mechanically ventilated patients with diffuse alveolar damage (DAD) on transbronchial biopsy (A, B), other types of lung injury on surgical lung biopsy (C, UIP-usual interstitial pneumonia; D, OP-organizing pneumonia; E, aspiration pneumonitis), or no specific pathology (F). Photomicrographs of P-S6 (Ser235/236) stained lung tissue obtained by transbronchial biopsy from patients with DAD (G-I), cryptogenic organizing pneumonia (COP, J), sarcoidosis (K), or no specific pathology (L). Black bar=3 mm.

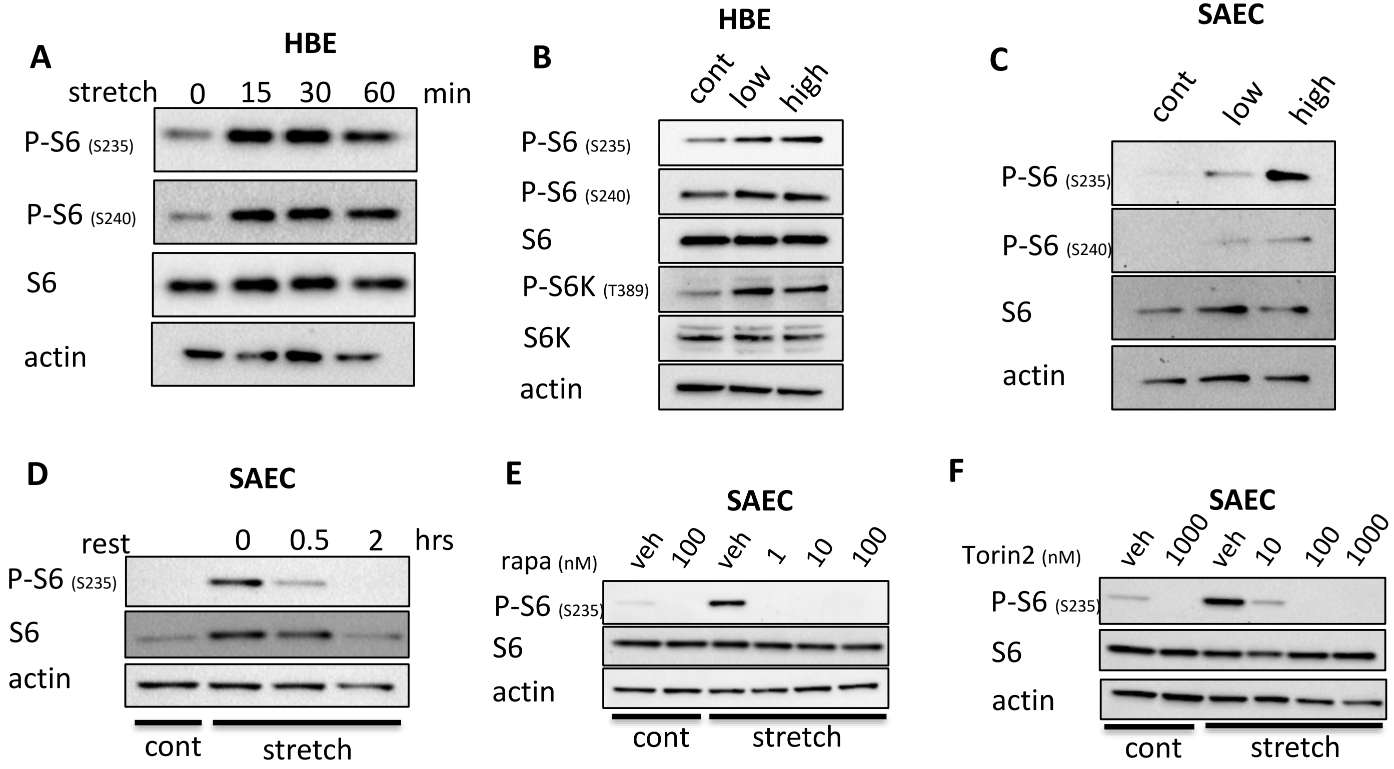

Supplemental Figure 9. Volutrauma rapidly activates mTORC1 in a dose dependent fashion. A) Human bronchial epithelial cells (HBE) were subjected to equibiaxial stretch (20%, 0.2 Hz) for varying amounts of time prior to immunoblotting for markers of mTORC1 activation (protein pooled from n=3 wells/lane). B) Human bronchial epithelial cells (HBEs) were subjected to 12% biaxial stretch (low) or 24% biaxial stretch (0.3 Hz) or static culture as control (cont) for 30 min prior to immunoblotting for markers of mTORC1 activation (protein pooled from n=6 wells/lane). C) Small airway epithelial cells (SAECs) were subjected to 12% biaxial stretch (low) or 24% biaxial stretch (0.3 Hz) for 4 hours prior to immunoblotting for markers of mTORC1 activation (protein pooled from n=6 wells/lane). D) SAECs were subjected to injurious biaxial stretch (24% stretch, 0.5 Hz, 4 hours) and then placed back into static culture (rest) for varying amounts of time prior to immunoblotting for markers of mTORC1 activation. (protein pooled from n=6 wells/lane) E) SAECs were subjected to in vitro volutrauma or static culture in the presence of increasing concentrations of rapamycin (rapa) for 4 hours prior to immunoblotting for markers of mTORC1 activation (protein pooled from n=3 wells/lane) F) SAECs were subjected to in vitro volutrauma or static culture in the presence of increasing concentrations of Torin 2 for 4 hours prior to immunoblotting for makers of mTORC1 activation (protein pooled from n=3 wells/lane).

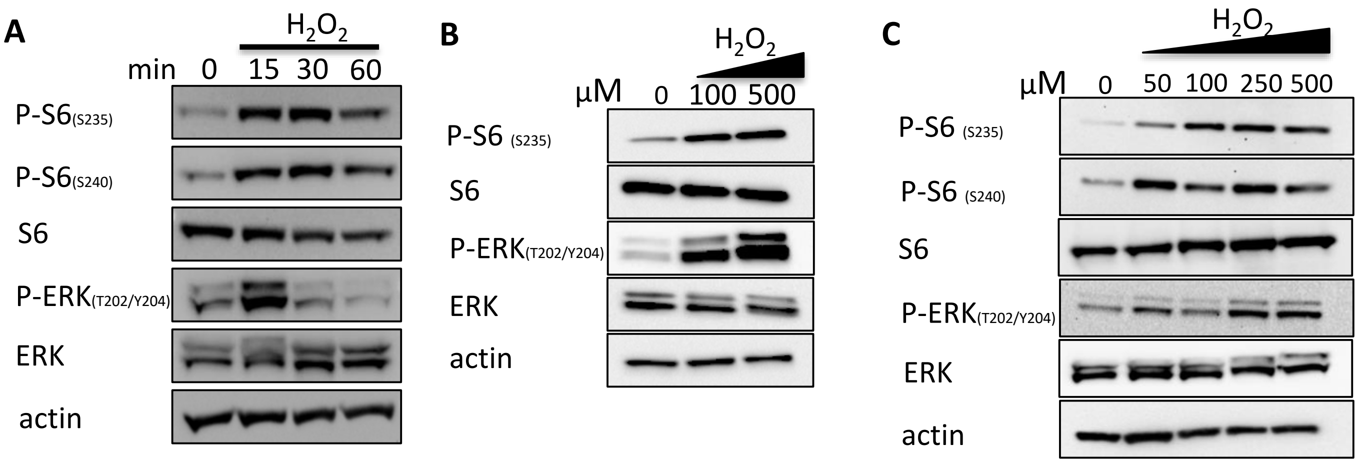

Supplemental Figure 10. Hydrogen peroxide rapidly activates mTORC1 in airway epithelial cells in a dose dependent fashion. A) Human bronchial epithelial cells (HBEs) were treated with 100 μM hydrogen peroxide (H_2O_2) for increasing amounts of time prior to immunoblotting for markers or ERK and mTORC1 activation. (protein from n=3 wells/lane) B) HBEs were treated with increasing doses of H_2O_2 for 30 min prior to immunoblotting for markers of ERK and mTORC1 activation. (protein from n=3 wells/lane) C) Small airway epithelial cells (SAECs) were treated with increasing doses of H_2O_2 for 30 min prior to immunoblotting for markers of ERK and mTORC1 activation. (protein from n=1 well/lane)

A

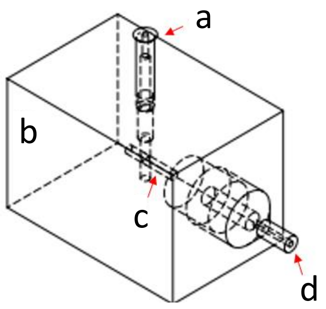

B

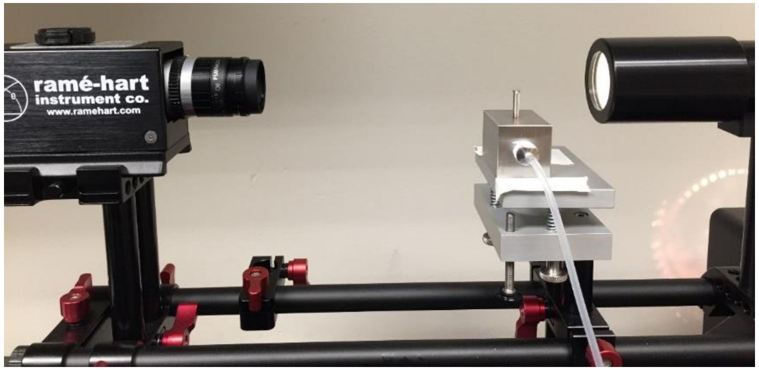

C

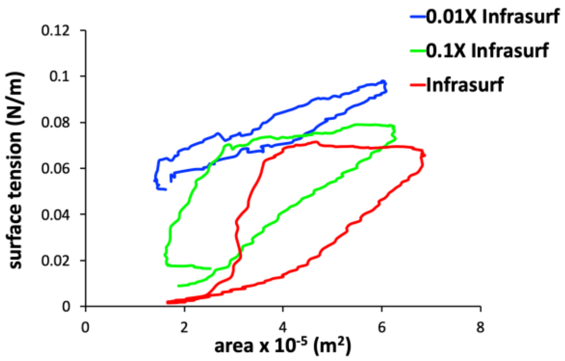

Supplemental Figure 11. Constrained drop surfactometer used to measure surfactant function. A) Constrained drop surfactometer (CDS) fabrication schematic. Droplet stage (a) is threaded into pedestal (b), with interior channels (c) continuous with syringe pump tubing port (d). B) CDS is placed upon goniometer stage for droplet recording. C) Surface tension vs surface area plots of serial dilutions of Infrasurf measured by surfactometry.

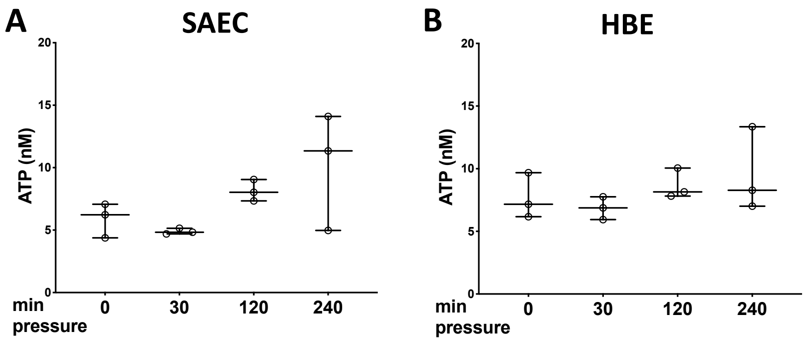

Supplemental Figure 12. In vitro barotrauma does not increase extracellular ATP release in airway epithelial cells. Small airway epithelial cells (SAEC, panel A) and human bronchial epithelial cells (HBE, panel B) were subjected to in vitro barotrauma (30 cm H_2O, 0.2 Hz) for varying amounts of time prior to measuring extracellular ATP. (n=3 wells/timepoint) Box blots show median +/- interquartile range and whiskers define min and max values.
